# CK2.1 Activates Chondrogenesis by Regulation of the p38 Mitogen-Activated Protein Kinase Pathway

**DOI:** 10.3390/kinasesphosphatases4020016

**Published:** 2026-06-15

**Authors:** Venu Pandit, Luke Fracek, Md Tamzid Hossain Tanim, Aarushi Patel, Daniel Halloran, Anja Nohe

**Affiliations:** Department of Biological Sciences, University of Delaware, Newark, DE 19711, USA

**Keywords:** chondrogenesis, BMP signaling, peptide drugs, disease-modifying OA drugs

## Abstract

Osteoarthritis (OA) remains a challenging disease due to the increased rate of incidence in the older population and the lack of a disease-modifying drug. BMP signaling plays a crucial role in chondrogenic differentiation and in the stability of articular cartilage. However, because BMP-2 also induces chondrocyte hypertrophy, it is not a viable drug for OA treatment. In contrast, the Bmpr1a biomimetic peptide can repair articular cartilage without inducing chondrocyte hypertrophy in the OA mouse model and in chondrocytes derived from patients diagnosed with OA. Despite this benefit, the mechanism by which the peptide drives chondrogenesis remains elusive. To explore this, we use a phosphoproteomics approach to identify pathways differentially activated by CK2.1. Specifically, we identified differentially phosphorylated phosphosites by CK2.1. Based on these phosphosites that we identified, we propose a molecular mechanism by which CK2.1 activates chondrogenesis. Notably, we predict that the mitogen-activated protein kinase (MAPK) pathway is regulated by CK2.1 to induce proteoglycan synthesis in C3H10T1/2 cells.

## 1. Introduction

Spontaneous or age-induced Osteoarthritis (OA) is a debilitating disorder of weight-bearing joints in the elderly. Weight-bearing joints become stiff due to inflammation, causing immobility and pain. Articular cartilage within the joints loses regenerative capacity [1]. People above the age of 65 are mostly affected, and women are more susceptible than men [1,2]. Popular treatment options are joint replacement surgery, and non-surgical options include intra-articular administration of NSAIDs and corticosteroids for pain relief [3]. However, despite these treatments, OA is not entirely prevented, as the progression of joint damage is not prevented [2,3].

Articular cartilage acts as a shock absorber and reduces friction during joint movement. Chondrocytes are the only cell type found within articular cartilage, constituting 10% tissue volume [4]. The remaining tissue volume is occupied by the extracellular matrix, composed of collagens, proteoglycans, and hyaluronan [4]. In healthy AC, chondrocytes are non-proliferative and nearly metabolically inactive [5]. Although they can synthesize the extracellular matrix of cartilage when articular cartilage is damaged [6]. In early OA, the metabolic activity of chondrocytes increases, specifically the cartilage synthesis pathways [7]. However, in severe OA, chondrocyte activity is dysregulated, driven by high levels of proteolytic enzymes [8]. Two main growth and differentiation factors—Bone Morphogenic Proteins (BMPs) and Transforming Growth Factor-β (TGF-β) regulate the homeostasis of the joint.

**Figure 1.**
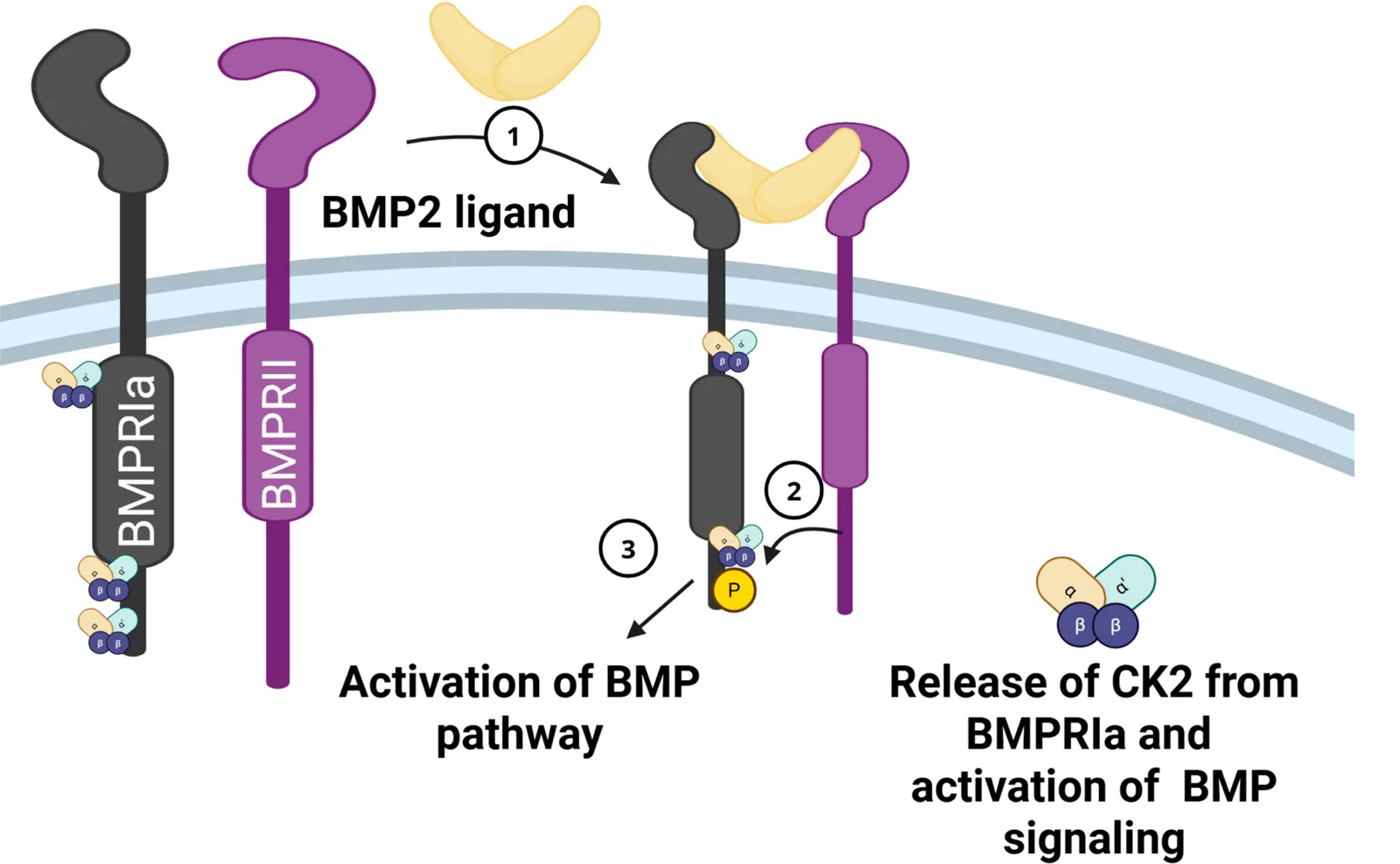
Activation of BMP signaling by BMP-2 and release of protein kinase II (CK2). In the absence of BMP2 ligand, the cytoplasmic domain of the BMPRIa receptor is associated with a pleiotropic kinase CK2. Upon binding BMP2 (1), the BMPRIa receptor heterodimerizes with BMPRII (2). The BMPRII receptor is a constitutively active kinase that phosphorylates BMPRIa (shown by arrow). This interaction of the BMP2 ligand with the BMPRIa-BMPRII complex leads to the release of CK2 (3) and activation of the BMP pathway. “Created in Biorender. Pandit V. (2026) https://BioRender.com/blwddx0”.

The bone morphogenetic protein signaling pathway is essential for the activation of cartilage synthesis in articular chondrocytes [9]. Chondrocytes express Bone Morphogenetic Protein Receptor Type Ia (Bmpr1a), which, upon binding BMP-2, activates BMP signaling by signal transduction (Figure 1). The binding of BMP-2 to Bmpr1a releases protein kinase 2 (CK2). BMP2 activates chondrogenesis and helps maintain chondrocyte phenotype in adult articular cartilage [10]. However, BMP-2 also drives chondrocyte hypertrophy. The process of chondrocyte hypertrophy, although essential during chondrocyte maturation and bone formation, is undesirable for articular cartilage repair in OA [11].

**Figure 2.**
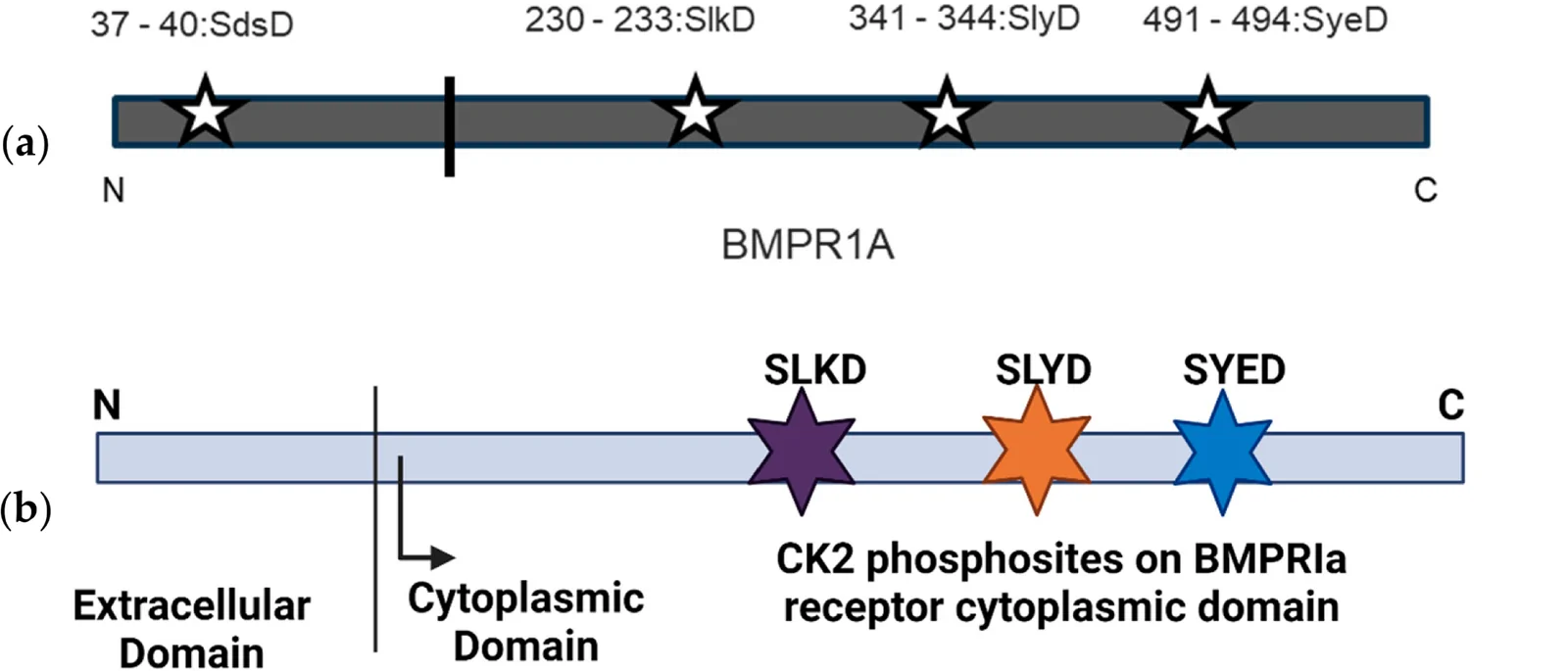
CK2 phosphorylation sites in the human Bmpr1a receptor sequence. (**a**) The CK2 phosphorylation motif S-XX-D/E is present at four positions in the human Bmpr1a receptor at positions 37, 230, 341, and 491 (denoted by stars). The vertical bar separates intracellular and extracellular domains; (**b**) the intracellular domain contains three of those sites-SLKD, SLYD, and SYED (denoted by stars).

A peptide drug, CK2.1, drives chondrogenesis while preventing chondrocyte hypertrophy in the DMM mouse model. However, the underlying molecular mechanism is unknown. The peptide is a Bmpr1a mimetic peptide containing the CK2 phosphorylation site ‘SYED’ flanked by an amino acid sequence near the site for sequence homology (Figure 2) and is proposed to prevent interaction between Bmpr1a and CK2 [12]. CK2.1 upregulates the expression of Collagen type II and type IX while downregulating the expression of Collagen Type X and MMP13 in an OA mouse model. However, the molecular mechanism by which CK2.1 induces cartilage synthesis in articular cartilage remains unclear.

**Figure 3.**
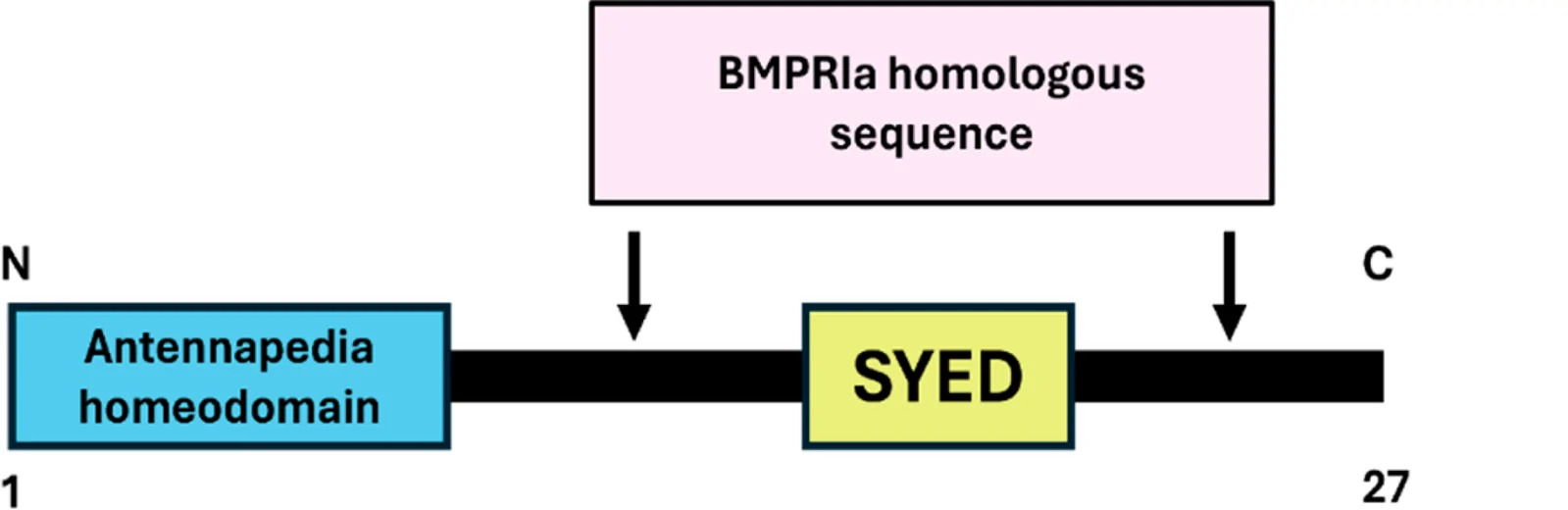
BMPR1a mimetic peptide CK2.1. Peptide CK2.1 is a 27-amino acid-long peptide with Antennapedia homeodomain at the N terminus and human BMPR1a sequence containing CK2 phosphorylation site SYED, along with nearby amino acids for sequence (in the regions indicated by arrows).

CK2.1 (Figure 3) increases the activity of the Smad-binding element, as with BMP-2 [13]. SMAD pathway activation by BMP signaling occurs during chondrocyte hypertrophy [14]. Bmpr1a can activate both canonical and non-canonical signaling [15]. Thus, CK2.1 may be upregulating SMAD-independent signaling. Thus, we hypothesized that CK2.1-mediated activation of cartilage synthesis depends on the regulation of non-canonical BMP signaling.

We used a phosphoproteomics approach to unravel the phosphorylation profile of CK2.1. In combination with differential phosphorylation of total proteins, we aim to predict the molecular targets of CK2.1. Using molecular pathway analysis tools, we predict the pathways that CK2.1 targets. In this study, we first characterized the differentially phosphorylated proteins between CK2.1 and BMP2. We then analyzed the significance of BMP2 pathway activation, both canonical and noncanonical, in CK2.1-driven chondrogenesis. Through this analysis, we predicted that CK2.1-induced chondrogenic activation depended on p38α activity. Finally, we inhibited p38α activity in C3H10T1/2 cells and assessed proteoglycan synthesis. We have thus proposed a new mode of chondrogenic activation of CK2.1, distinct from the Smad1-activated pathways induced by BMP2.

## 2. Results

### 2.1. Phosphoproteomics Analysis Revealed Differentially Phosphorylated and Total Proteins in CK2.1 and BMP2-Treated C3H10T1/2 Cells

We compared total protein phosphorylation and expression in C3H10T1/2 cells treated with CK2.1 and BMP2. Unstimulated cells were used as a control. Prior studies by Ali et al. (2022) [16] and Hsueh et al. (2016) [17], which performed the proteomics analysis of cartilage/chondrocytes and used *p*-values and non-zero-fold changes. The same approach was used to develop the data analysis pipeline in the present study. This approach helps us analyze a larger number of phosphosites to predict the mechanism of CK2.1. A total of 5529 phosphosites were analyzed. Between CK2.1 and Unstimulated, there were a total of 57 phosphosites, whereas between BMP2 and Unstimulated, there were a total of 32 phosphosites differentially phosphorylated (*p* < 0.05). Between CK2.1 and BMP2-treatment groups, 23 phosphosites were phosphorylated significantly differently (*p* < 0.05) (Supplementary Materials Figure S1). CK2.1 caused differential expressions of 8 proteins, BMP2 caused differential expression of 6 proteins, and between BMP2 and CK2.1, there was one protein differentially expressed (fold change > 1 or <−1 with a *p* value <0.05). A total of 5352 proteins were analyzed.

**Figure 4.**
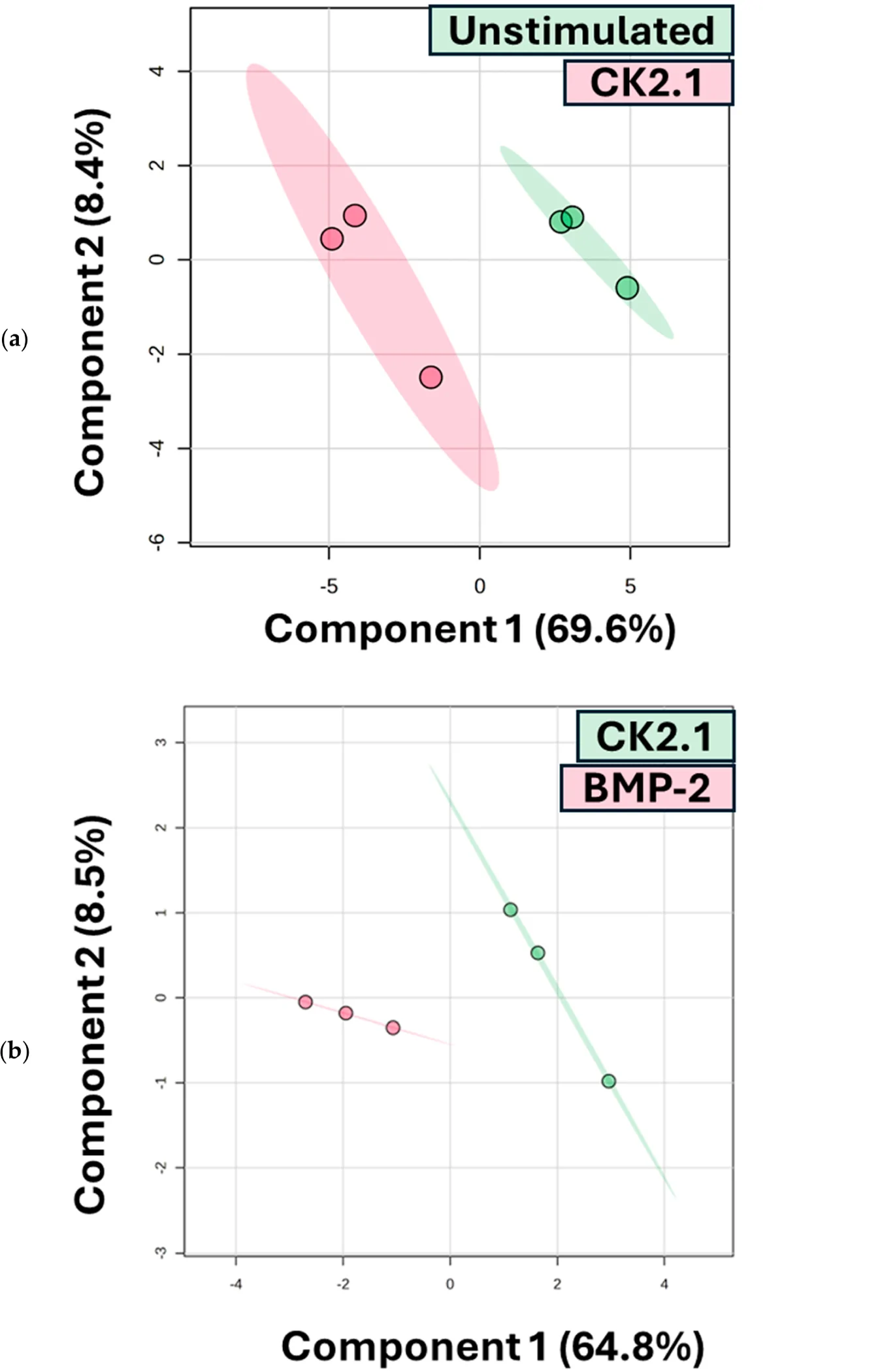

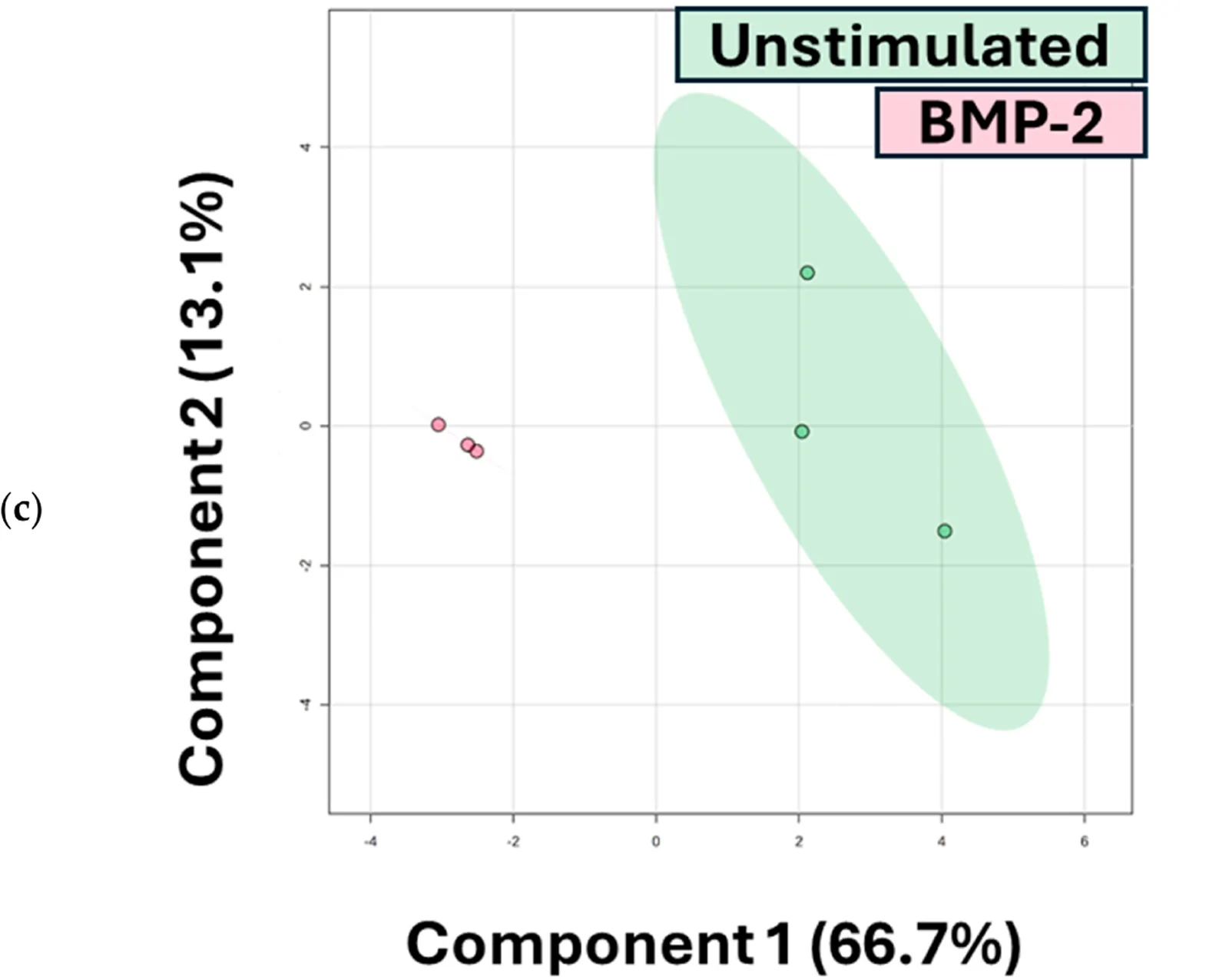
Supervised multivariate analysis (PLS-DA) was performed on differentially phosphorylated phosphosites (*p* < 0.05). (**a**) CK2.1-treated and Unstimulated samples (*n* = 3 per group), and scores are plotted on components 1 versus 2, which explain 69.6% and 8% of the variance, respectively. (**b**) CK2.1-treated and BMP-2-treated samples (*n* = 3 per group), and scores are plotted on components 1 versus 2, which explain 64.8% and 8.5% of the variance, respectively. (**c**) Unstimulated and BMP-2 treated samples (*n* = 3 per group), and scores are plotted on components 1 versus 2, which explain 64.8% and 8.5% of the variance, respectively. The model shows clear separation between BMP-2–treated and CK2.1-treated samples, indicating distinct phosphorylation profiles. Colors indicate treatment groups and ellipses represent 95% confidence regions. Dots indicate individual samples.

We performed supervised clustering using Partial Least Squares Discriminant Analysis (PLS-DA) on all differentially phosphorylated phosphosites. Based on clustering, the BMP2-activated and CK2.1-activated phosphoproteomes show a robust distinction (Figure 4). The strong positive loadings (Supplementary Data Figure S1) in the CK2.1 phosphoproteome corresponded to phosphosites on proteins Rbm10, Nol8, Histone H4, Nop14, Zc3h18, and Bclaf. Component 1 accounts for 64% of the variance in the differential phosphosites. Nol8, Nop4, and Rbm10 have significant fold change (Log FC > 1; *p* < 0.05). These are nuclear or nucleolar proteins. Nol8 binds the β-catenin protein in the nucleus. The binding of Nol8 results in the modulation of canonical Wnt/β-catenin-regulated gene expression [18]. The Wnt/β-catenin pathway is essential for cartilage repair post-injury [19]. Nop14 enhances mTORC2 activity [20]. The mTORC2 complex formation is essential for the mechanical stress response of cartilage. The pathway also increases chondrocyte survival [21]. Rbm10 is a nucleolar RNA-binding protein. It regulates splicing of DNA (cytosine-5)-methyltransferase 3b (Dnmt3b). The Dnmt3b enzyme regulates expression at NF-κB-regulated promoters. Thus, Rbm10 modulates activation of NF-κB-responsive genes [22]. The NF-κB signaling is essential in the expression of catabolic proteins in cartilage, such as Mmp13 [23].

Comparison of phosphosites between BMP2 and Unstimulated resulted in a compact ellipse denoting BMP2 replicates, with a strong negative correlation in component 1, at 66% coverage. Between CK2.1 and Unstimulated, there is high variability between samples as compared to Unstimulated. Component 1 covers 69% variance. There are very few phosphosites, and the differential phosphosites between CK2.1 and BMP2 are limited. Yet, according to the PLS-DA analysis, phosphosites showed distinct phosphorylation patterns.

### 2.2. Identified Phosphosites Are Differentially Phosphorylated in the CK2.1 and BMP2-Treated Mouse Model

**Figure 5.**
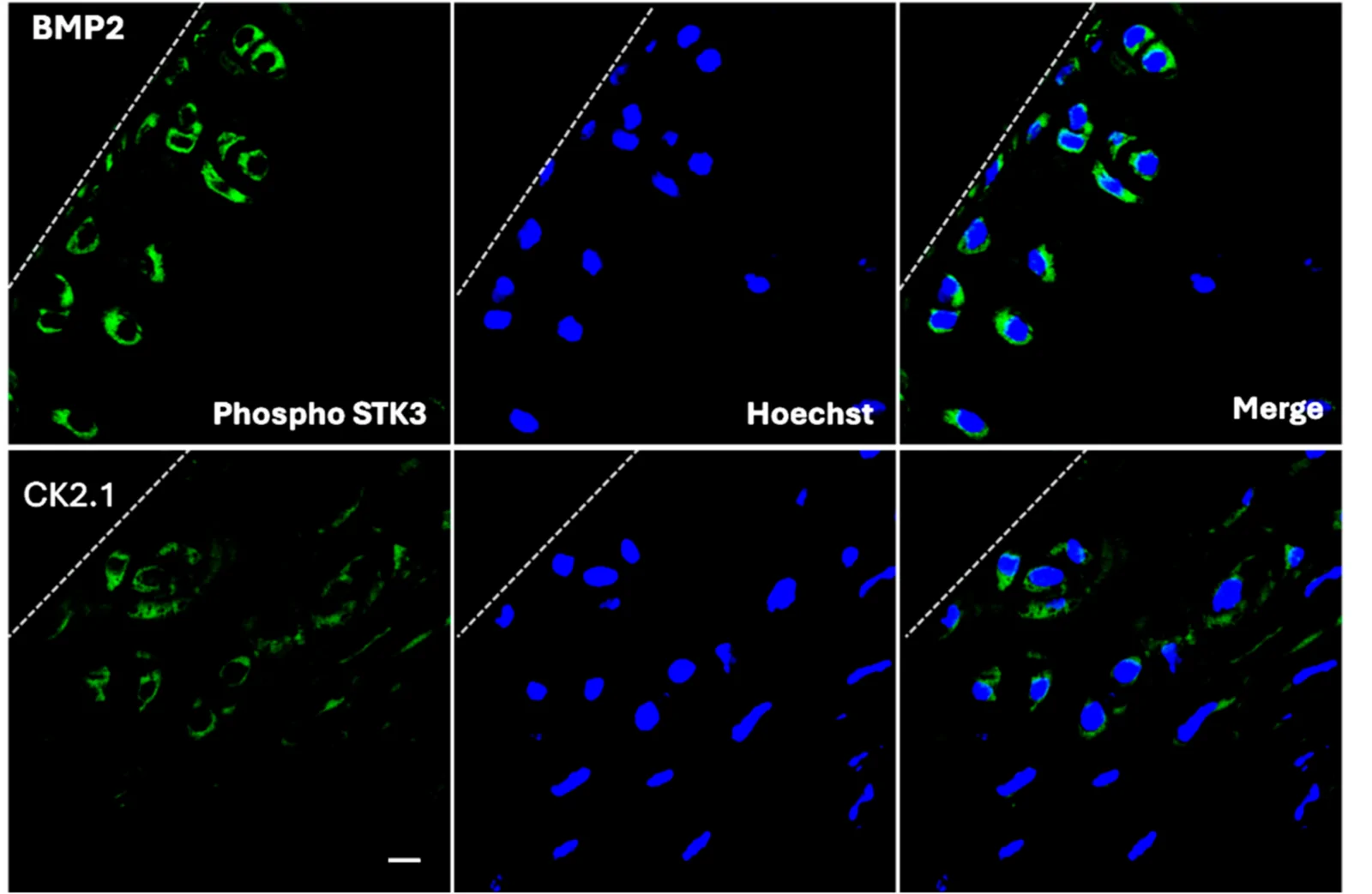
Validation of differential phosphorylation using immunofluorescence. Phosphorylation of STK3 T174 (green) was downregulated in CK2.1-treated samples compared to BMP-2-treated samples (from phosphoproteomics analysis log FC = −1.4, *p* < 0.05). Immunofluorescence detection of STK3 phosphorylation at T174 using a phosphorylation-specific antibody showed reduced STK3 phosphorylation in systemic CK2.1-injected mice vs. BMP-2-injected mice. (scale bar-10 μm).

**Figure 6.**
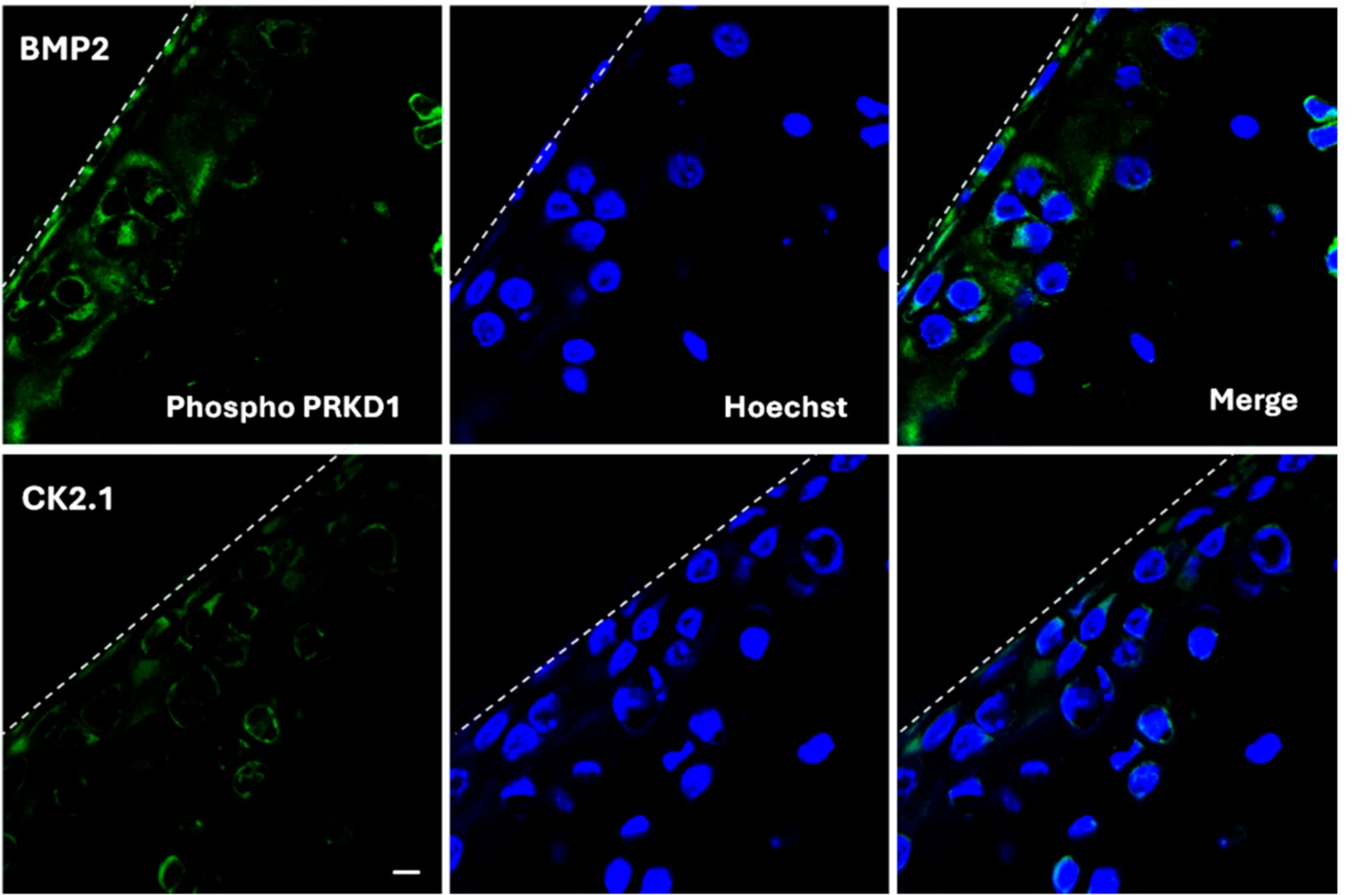
Validation of differential phosphorylation using immunofluorescence. Phosphorylation of PRKD1 was downregulated in CK2.1-treated samples compared to BMP-2-treated samples (from phosphoproteomics analysis log FC = −1.2, *p* < 0.05). Immunofluorescence detection of PRKD1 phosphorylation at T752 using a phospho-specific antibody showed reduced PRKD1 T752 (green) phosphorylation in systemic CK2.1-injected mice vs. BMP-2-injected mice. (scale bar-10 μm).

After identification of significantly differentiated phosphosites, we validated their differential phosphorylation. We selected the phosphosites Serine threonine kinase 3 (Stk3) T174 and protein kinase D1 (Prkd1) T752 because they exhibited the highest magnitude of fold change (Stk3 log_2_FC −1.4 and Prkd1 log_2_FC 1.2, *p* < 0.05 from phosphoproteomics data) in phosphorylation levels between CK2.1 and BMP2. The expression levels of Stk3 and Prkd1 did not differ significantly between the control and BMP2 groups. Systemic injection of CK2.1 results in upregulation of chondrogenic markers, including Collagen type II and Collagen type IX. CK2.1 downregulates chondrocyte hypertrophy markers, such as Mmp-13 and Collagen Type X [13]. We used the same system because CK2.1 induced chondrogenesis in C57BL/6 mice when injected systemically [24]. Mice injected with PBS were used as controls. Here, we observed downregulation of Stk3 (Figure 5) and Prkd1 (Figure 6) phosphorylation in immunofluorescence. Serine/threonine-protein kinase 3/4 (Stk3/4) kinases belong to the Hippo-YAP signaling pathway. These kinases phosphorylate YAP, preventing its nuclear localization [25]. Cytoplasmic localization of YAP proteins inhibits activation of osteogenic genes, such as Collagen Type X. Prkd1 is a downstream activator of the mTORC1 complex [26]. Deletion of mTORC1 rescues the OA phenotype in the DMM mouse model [27]. The mTORC1 complex regulates autophagy and apoptosis in chondrocytes [28].

### 2.3. Differentially Phosphorylated Proteins by CK2.1 Are Associated with Non-Canonical BMP Signaling

Next, we predicted associations between BMP signaling proteins and differentially phosphorylated proteins between CK2.1 and the untreated control (Unstimulated). (The comparison between CK2.1 and Unstimulated and CK2.1—BMP2 is provided in the Supplementary Materials (Figure S2). We compared the association of CK2.1 differentially phosphorylated proteins with the canonical BMP signaling pathway (Smad1-dependent) and the p38 MAPK pathway. The list of proteins involved in canonical BMP-2 signaling and p38 MAPK signaling was curated from the literature [15,28–31] (Supplementary Materials Table S2). BMP-2 activates several branches of non-canonical BMP signaling, including the EKR, PI3K, and Tab/Tak1 pathways [10]. The Tak1 pathway activates the JNK, ERK, and p38 MAPK pathways in chondrocytes [29]. Of these, the p38 pathway is essential for chondrogenic differentiation and for regulating hypertrophic differentiation [30–32]. The p38 MAPK pathway proteins were identified as differentially phosphorylated by CK2.1, including Mapk14 (p38α), Mapk1, and Map3k3. Thus, we compared the association of all the differentially phosphorylated proteins by CK2.1 with the p38 MAPK pathway. We used the STRING database (https://string-db.org/) to analyze differentially phosphorylated proteins by CK2.1. This database creates protein networks based on neighborhood chromosomes, gene fusions, phylogenetic co-occurrence, homology, coexpression, and experimentally determined interactions. It creates pathway enrichment analysis using the protein over-representation method [33].

**Figure 7.**
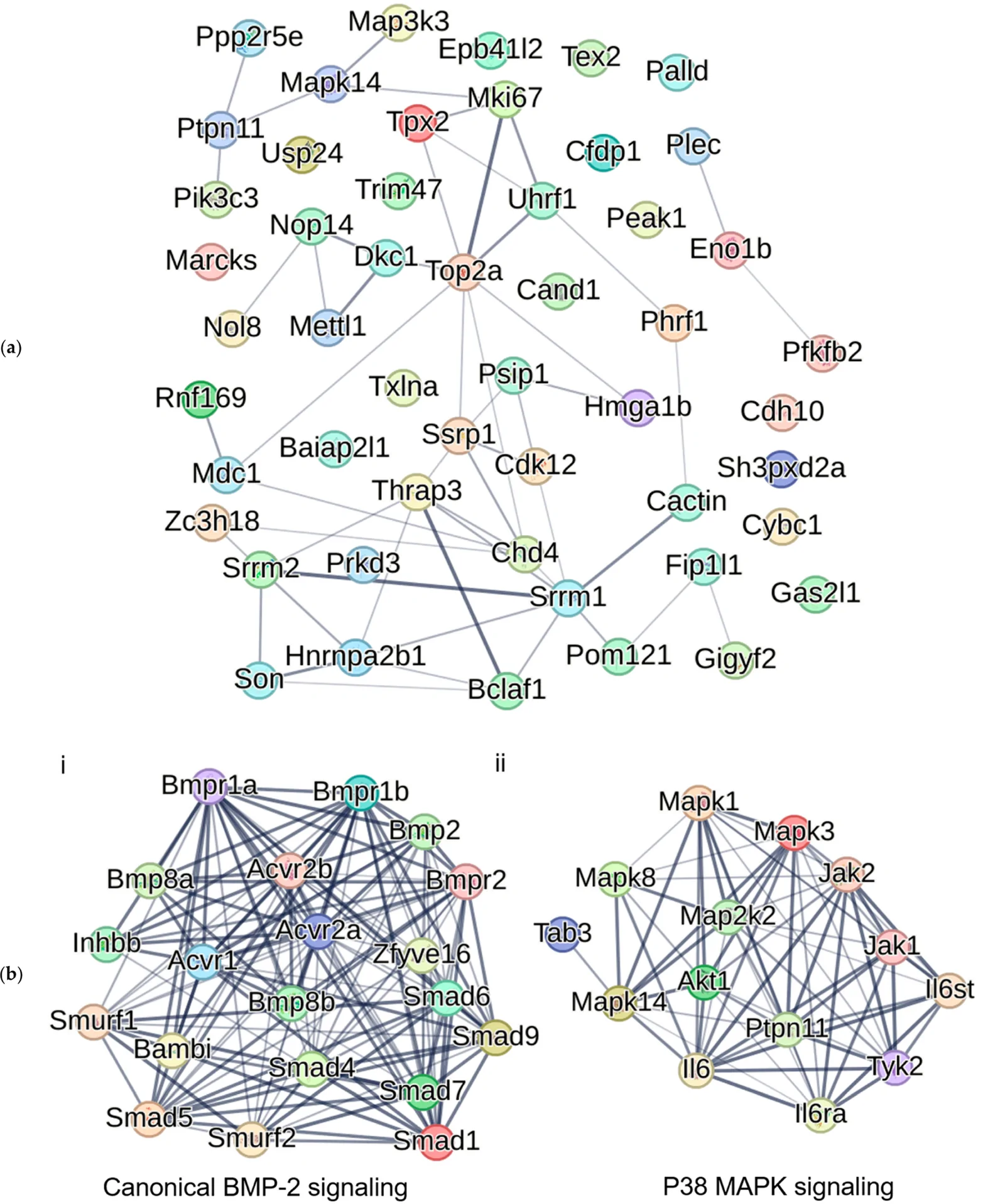

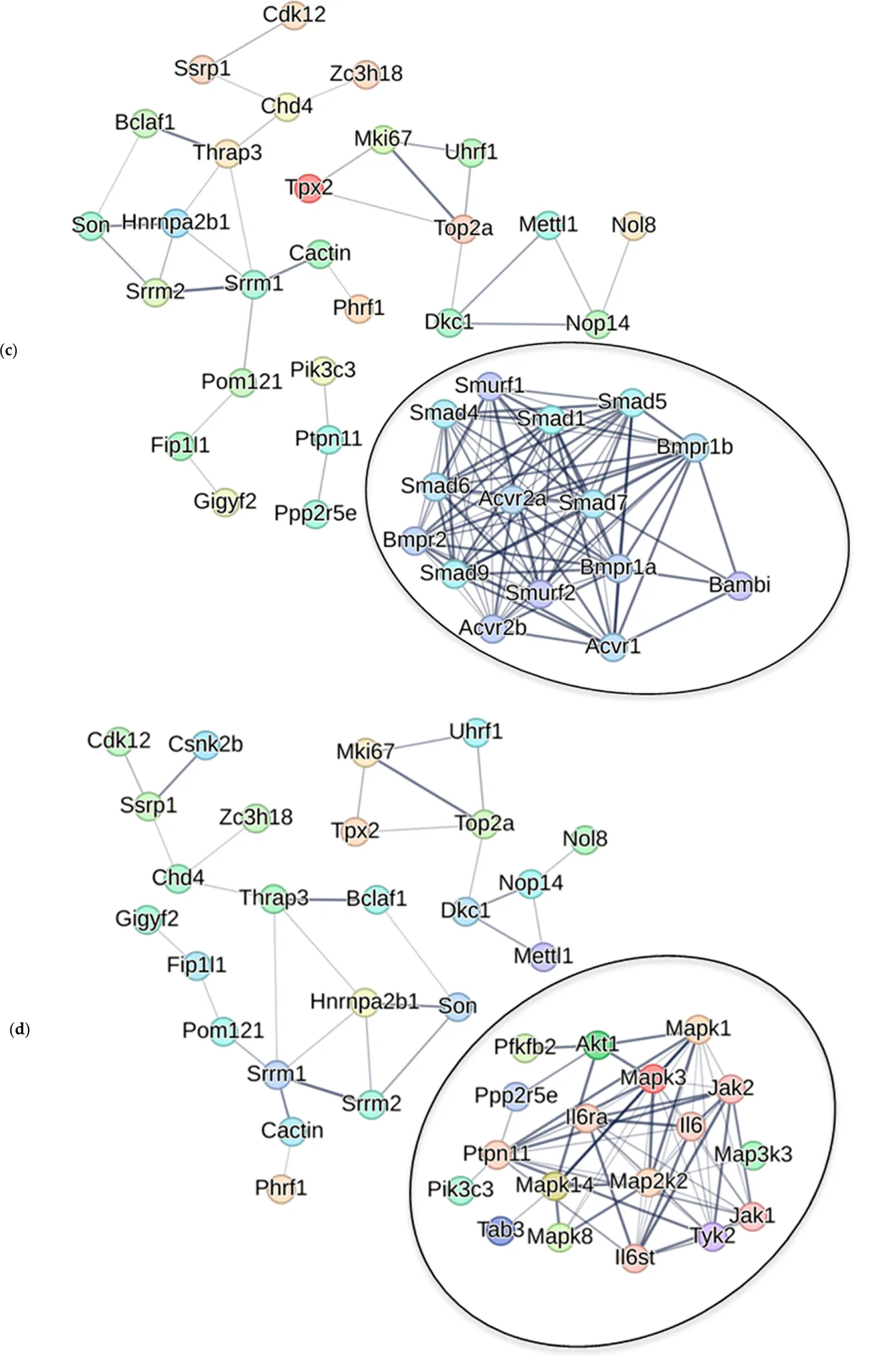
Analysis and visualization of CK2.1-driven differentially phosphorylated proteins in mouse mesenchymal C3H10T1/2 cells. (**a**) Interactions between differentially phosphorylated phosphosites by CK2.1 (**b**) STRING network of canonical BMP-2 (i) signaling and p38 MAPK (ii) signaling. (**c**) Proteins differentially phosphorylated by CK2.1 compared to Unstimulated were combined, and their association with canonical BMP signaling was examined. No association was found between the proteins and canonical BMP signaling. (**d**) Proteins differentially phosphorylated by CK2.1 compared to Unstimulated were combined, and their association with p38 signaling was examined. Few proteins are associated with p38 MAPK signaling. Colored solid lines indicate the confidence level of the interaction, with darker lines indicating greater confidence.

**Figure 8.**
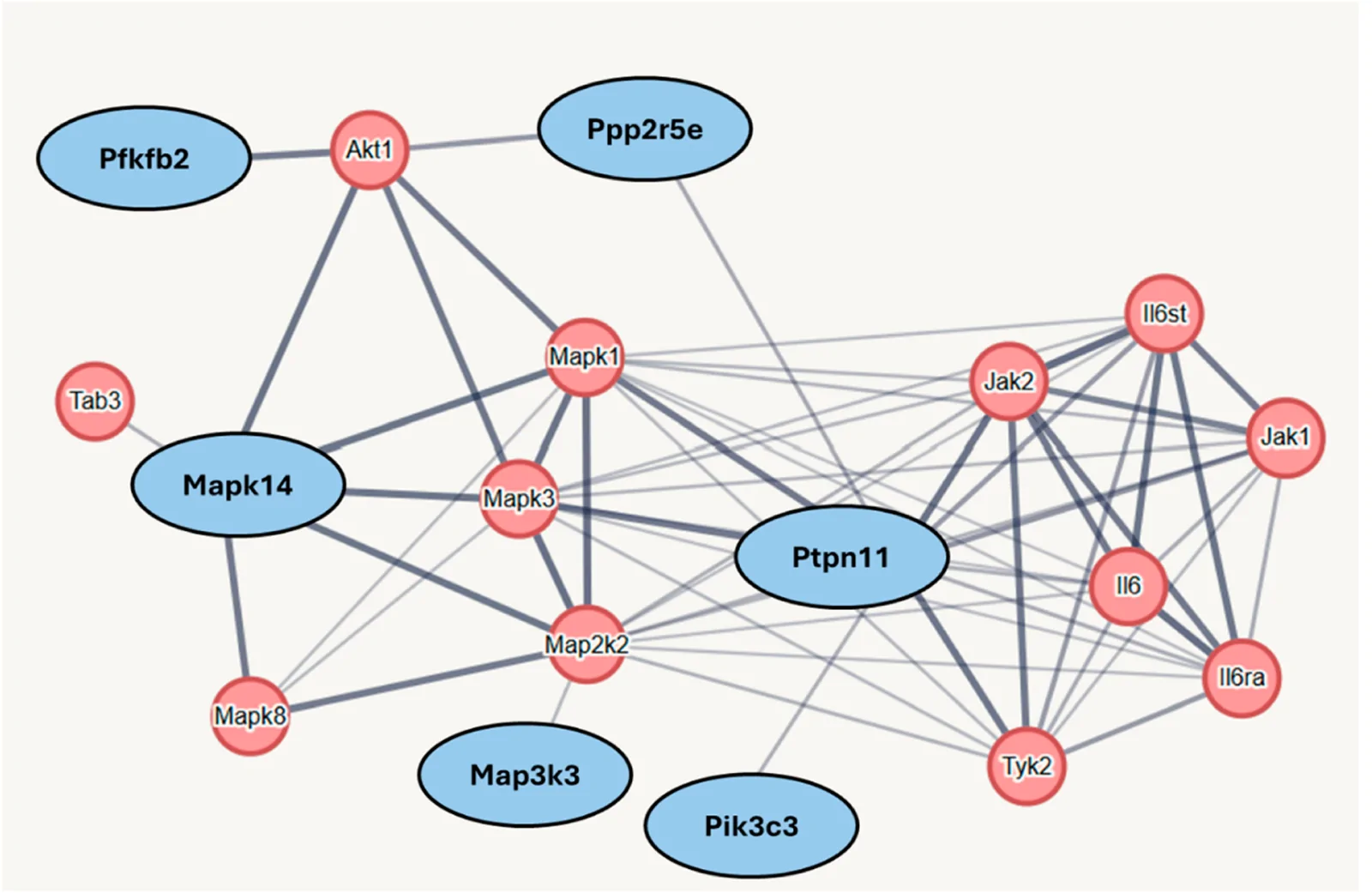
Association of differentially phosphorylated proteins by CK2.1 was associated with p38 MAPK pathway proteins. Network between differentially phosphorylated proteins by CK2.1 and p38 MAPK pathway proteins; proteins included Pfkfb2, Ppp2r5e, Mapk14, Ptpn11, Map3k3, Pik3c3 (highlighted in blue).

We visualized the protein phosphorylation network mediated by CK2.1 (Figure 7a). Further, we visualized the network between canonical BMP-2 signaling and p38 MAPK (Figure 7b) signaling components with these proteins. There was no link between canonical BMP signaling proteins and the phosphosites differentially phosphorylated by CK2.1 (Figure 7c). However, the p38 pathway proteins were associated with the differentially phosphorylated proteins (Figure 7d). The list includes—Pfkfb2 (6-phosphofructo-2-kinase/fructose-2,6-bisphosphatase 2;6-phosphofructo-2-kinase;Fructose-2,6-bisphosphatase), Map3k3, (mitogen-activated protein kinase kinase kinase 3), Pik3c3 (Phosphatidylinositol 3-kinase catalytic subunit type 3), Ppp2r5e (Serine/threonine-protein phosphatase 2A 56 kDa regulatory subunit epsilon isoform), Ptpn11 (Tyrosine-protein phosphatase non-receptor type 11) and Mapk14 (mitogen-activated protein kinase 14 or p38α) (Figure 8). Of these proteins, Map3k3, Mapk14, and Ptpn11 (log2Fc −1.2, −1, and 0.7, *p* < 0.05) are either MAPK or associated proteins. Ptpn11 is a positive regulator of the MAPK pathway. CK2.1 downregulated p38 Y182. This phosphosite shows a significant fold change (Mapk14 Log_2_FC = −1; *p* < 0.05) and significant loadings score in supervised clustering between CK2.1 and Unstimulated (Supplementary Materials Figure S1).

### 2.4. CK2.1 Phosphorylated p38 MAPK-Related Proteins in Mouse Bone Marrow Stromal Cells

Previously, OA was characterized as a disease of articular cartilage. Recent advances in understanding the pathological changes and underlying molecular mechanisms have established that different tissue compartments within the articular joint exhibit signs of OA onset [3,34,35]. The bone marrow compartment in the subchondral bone is deemed important in the upcoming model of spontaneous or age-associated OA progression [36]. This model emphasizes that the dysregulated and excessive remodeling of subchondral bone due to bone marrow lesions preclude OA-associated changes in the articular joint [37]. Bone marrow lesions enable migration of osteoclast precursors and secretion of inflammatory cytokines into articular cartilage. Moreover, the expression profiles of immune cells in the subchondral region are altered in patients with OA [38]. Thus, understanding the changes in molecular pathways differentially activated in the bone marrow microenvironment is essential for elucidating the pathology of spontaneous OA [39]. Bone marrow stromal cells have a major role in regulating the bone marrow microenvironment. Bone marrow stromal cells represent a heterogeneous population of cells with stem cell properties. BMP-2 can activate chondrogenesis in the BMSC-derived mesenchymal stem cell population (MSC) [40–42]. Thus, we analyzed the differential activation of cellular processes in BMSCs and their association with canonical BMP-2 signaling and p38 signaling.

**Figure 9.**
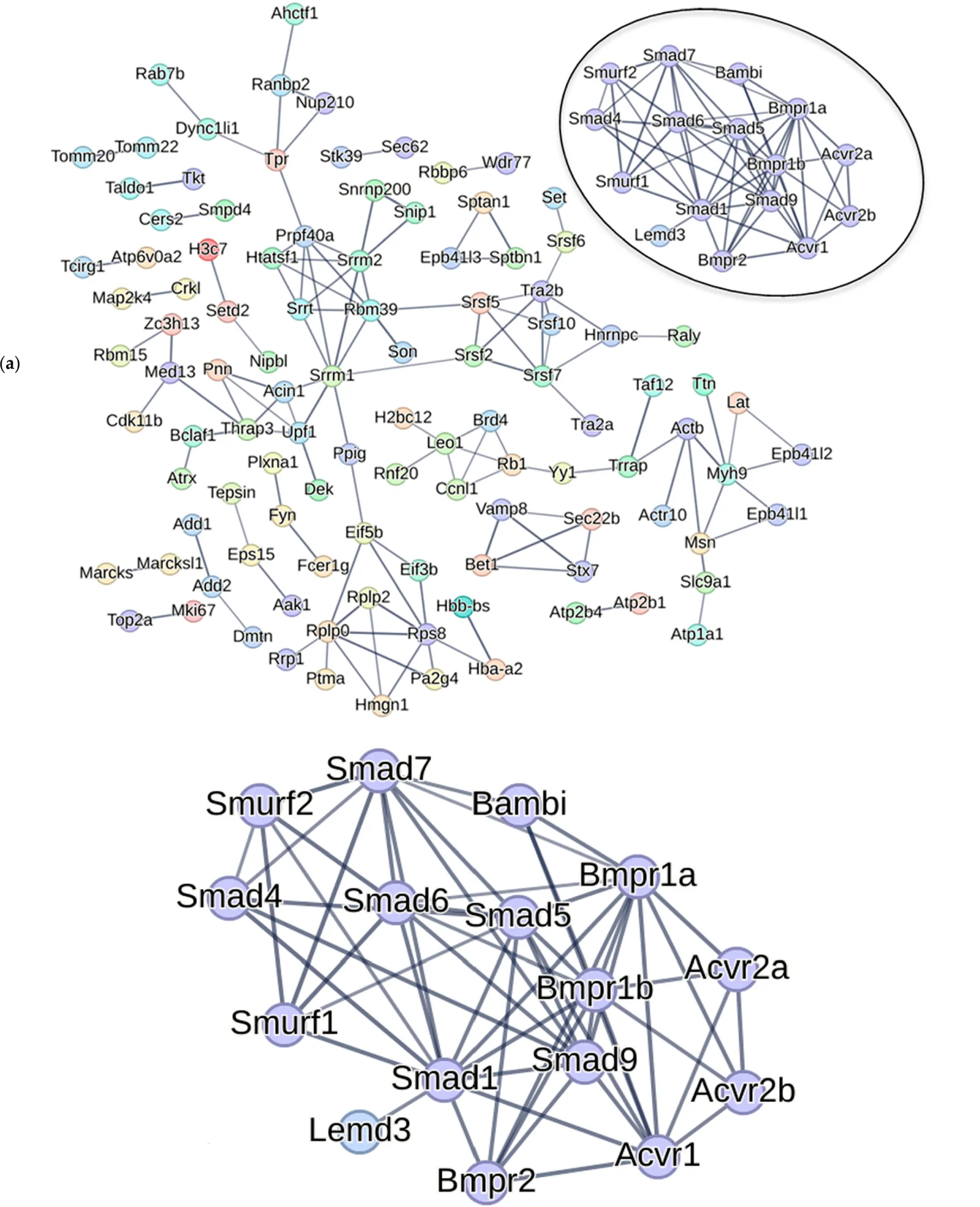

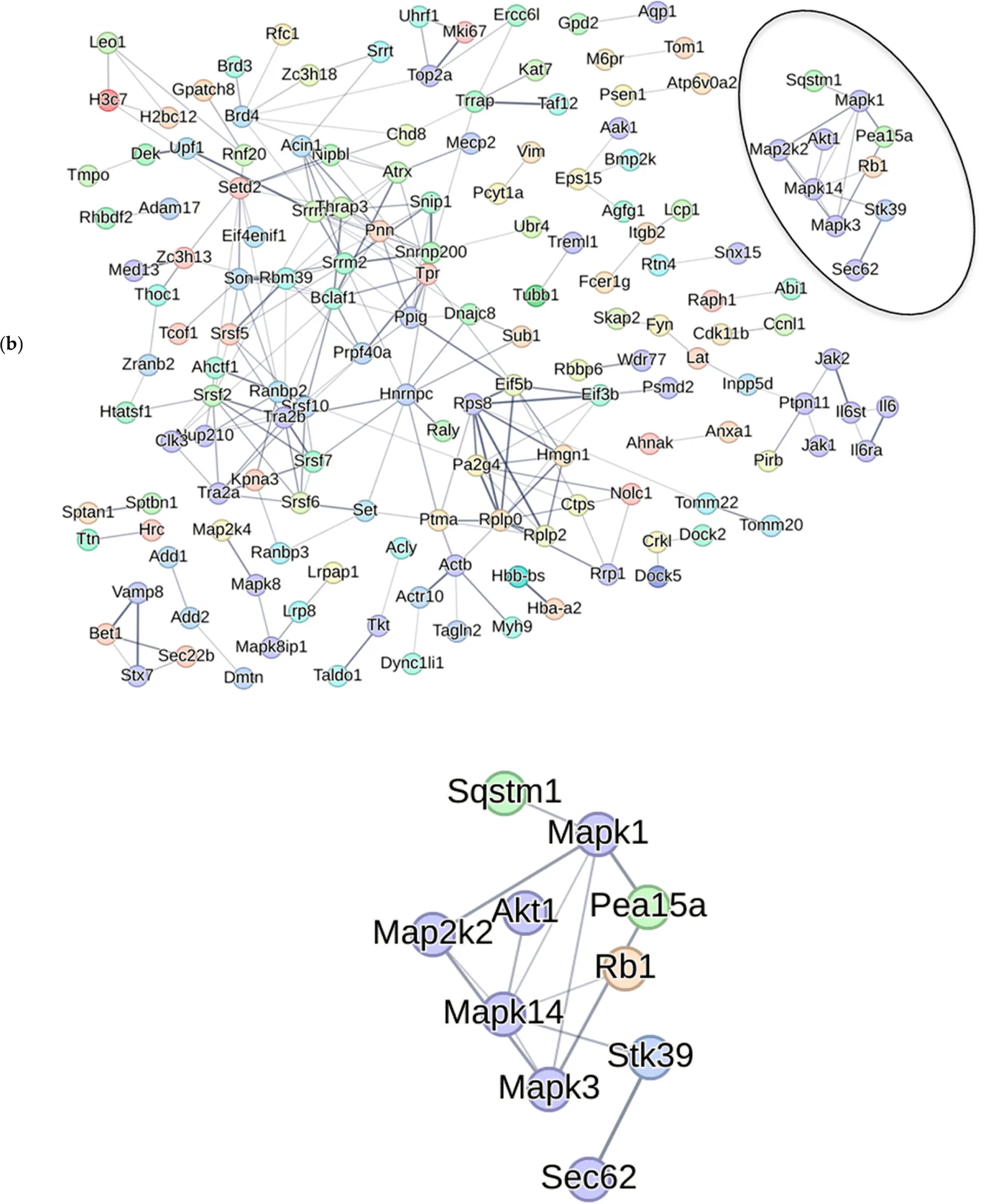

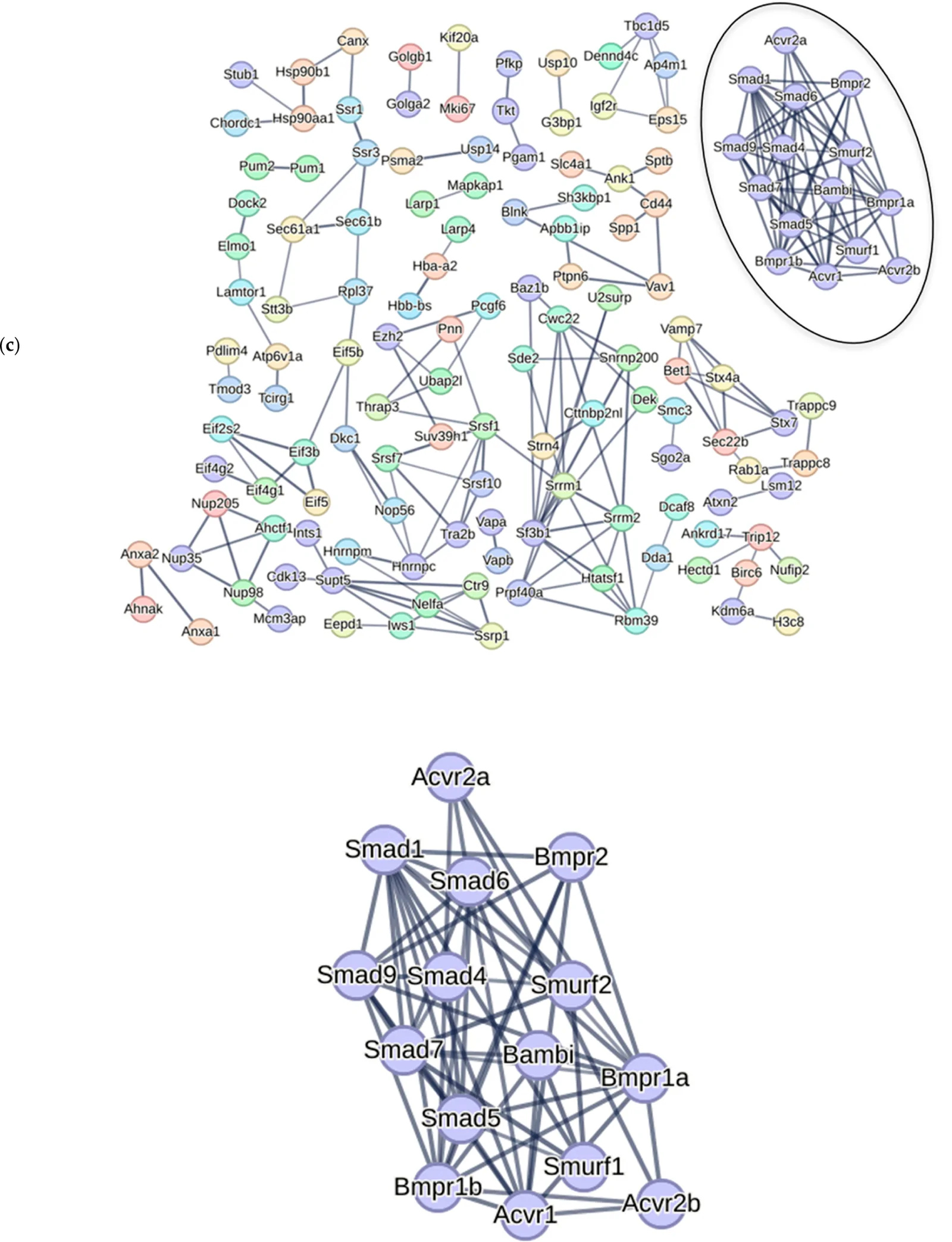

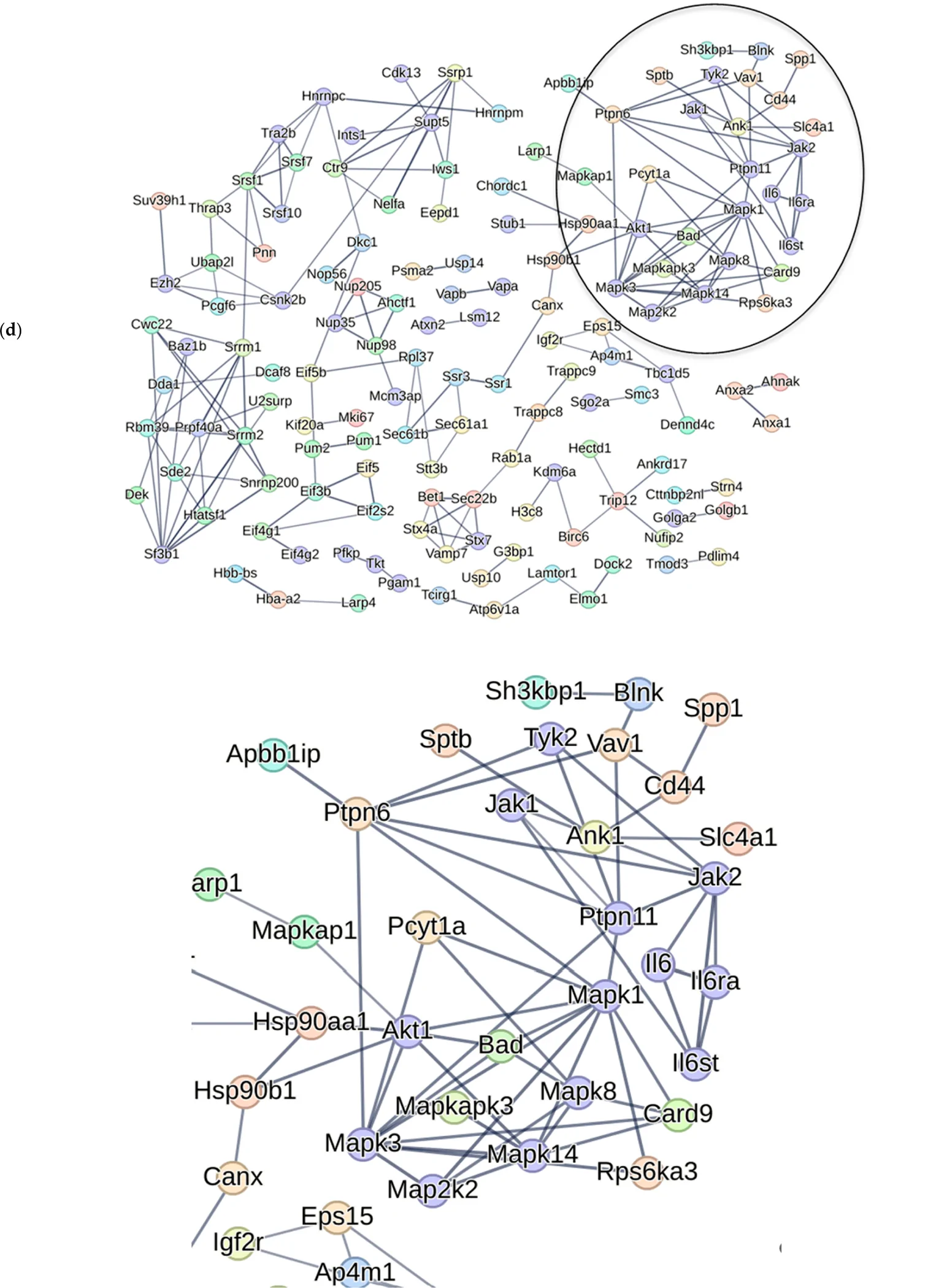
Analysis and visualization of CK2.1-driven differentially phosphorylated proteins in mouse bone marrow stromal cells. STRING network analysis showing association between CK2.1-driven differential phosphorylation of proteins in mouse BMSCs associated with (**a**) BMP-2 canonical signaling in 6-month-old mice; (**b**) p38 signaling in 6-month-old mice; (**c**) BMP-2 canonical signaling in 15-month-old mice; (**d**) p38 MAPK signaling in 15-month-old mice. Association between CK2.1-driven differential phosphorylated proteins associated with p38 MAPK signaling in BMSCs of 6 and 15-month-old mice. Colored solid lines indicate the confidence level of the interaction, with darker lines indicating greater confidence. The magnified images of a part of the network show interactions between differentially phosphorylated proteins and proteins of the BMP canonical pathway or the p38 MAPK pathway.

We treated BMSCs from 6- and 15-month-old C57BL/6 mice (n = 9 each) with CK2.1 in vitro. The 6-month-old mice represent a young population without spontaneous OA. C57BL/6 mice develop spontaneous OA with aging. Thus, 15-month mice represent a senile population with spontaneous OA [43,44]. We mapped the network of differentially phosphorylated proteins by CK2.1 in BMSCs from 6- and 15-month-old mice and performed Gene ontology enrichment using the Reac-tome database (Figure S3). The differential phosphoproteome of BMSCs was analyzed for its association with canonical BMP signaling and the p38 pathway. Here, as in C3H10T1/2 cells, the differentially phosphorylated proteins were not associated with canonical BMP-2 signaling (Figure 9a,b) but with the p38 MAPK pathway (Figure 9c,d).

### 2.5. The MAPK Pathway Plays a Key Role in Chondrogenic Activation

**Figure 10.**
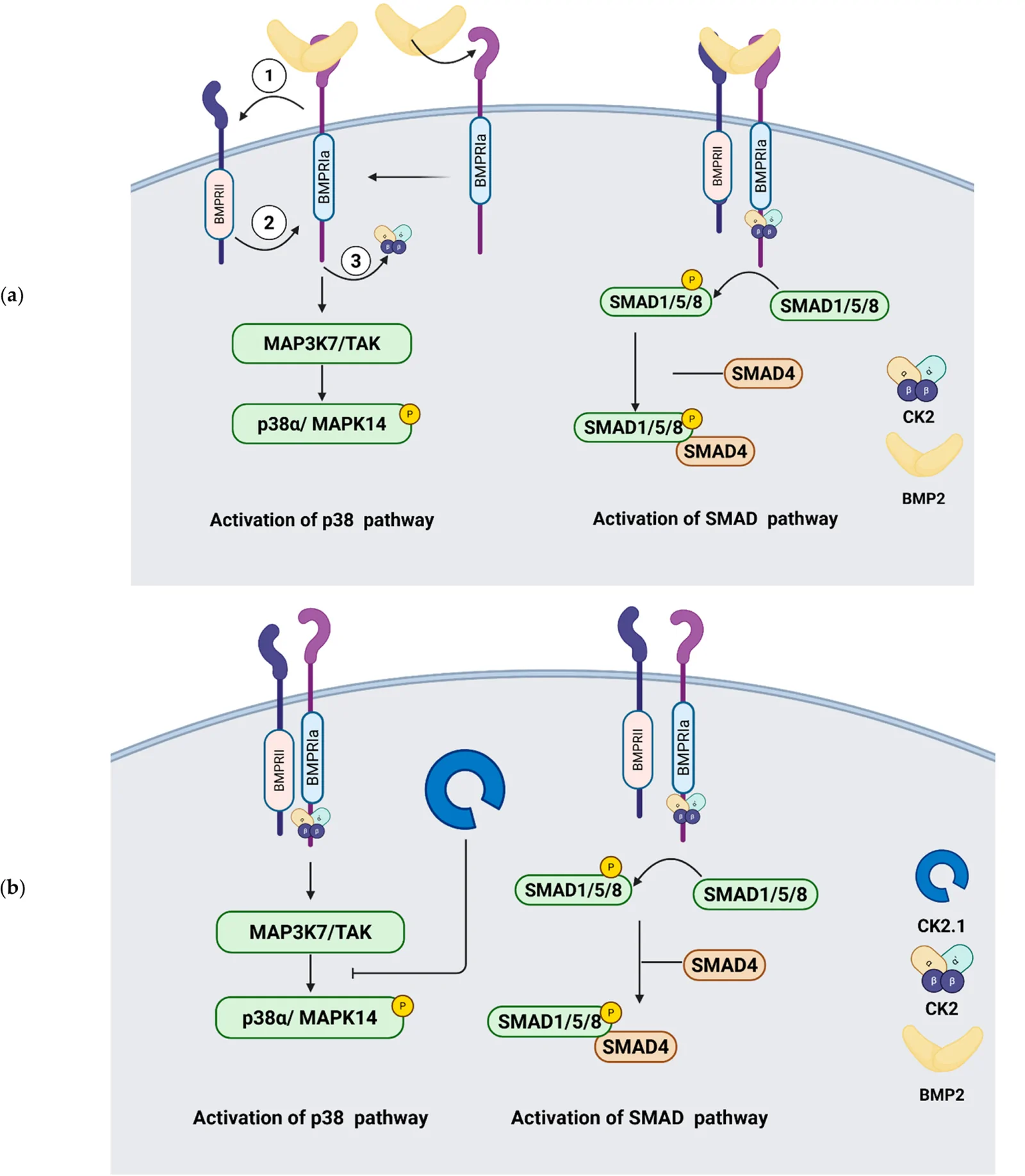
Regulation of p38 MAPK pathway by CK2.1. (**a**) BMP-2 binding with Bmpr1A causes its hetero-oligomerization (shown as a dimer for simplicity) with BMPR2 (1). The constitutively active kinase domain of BMPR2 activates the serine threonine kinase domain of Bmpr1a (2), and this causes the release of protein kinase CK2 from the Bmpr1a receptor (3). Activation of Bmpr1a by these events activates the p38 MAPK pathway. BMP-2 binding to pre-formed complexes of BMPR1a and BMPR2 activates the Smad phosphorylation pathway. Phosphorylation of p38 at Y182 is required for its activation. Activation of p38 in chondrocytes induces chondrocyte differentiation, resulting in endochondral ossification of the cartilage; (**b**) CK2.1 activates the BMP pathway without the involvement of the BMP-2 ligand. The proposed mechanism involves activation of the Bmpr1a receptor by the release of protein kinase CK2, which in turn activates the intracellular serine-threonine kinase. Phosphorylation of p38α at Y182 is downregulated in CK2.1-treated C3H10T1/2 cells. Arrows indicate activation. (Created in BioRender. Pandit, V. (2026) https://BioRender.com/759f4xb).

We then predicted a mechanism by which CK2.1 interacts with the MAPK pathway to activate chondrogenesis. The p38 MAP kinase plays an important role in regulating chondrogenesis. Early inhibition of p38 activates chondrogenesis [32], whereas in cells committed to a chondrogenic phenotype, p38α inhibition downregulates Sox9 activity and delays the expression of chondrogenic proteins such as Collagen type II [14,45,46]. Sox9 activation in mesenchymal stem cells promotes chondrogenesis [47]. The peptide CK2.1 is a biomimetic peptide of Bmpr1a and is predicted to inhibit the interaction between Bmpr1a and CK2 (Figure 10). Thus, rendering the intracellular BMP signaling active in the absence of BMP2 ligand. Here, the downregulation of p38α Y182 phosphorylation by CK2.1 could be priming mouse mesenchymal cells for chondrogenic activation.

### 2.6. Activation of Chondrogenesis by CK2.1 Depends on the Early Downregulation of p38α Activity

**Figure 11.**
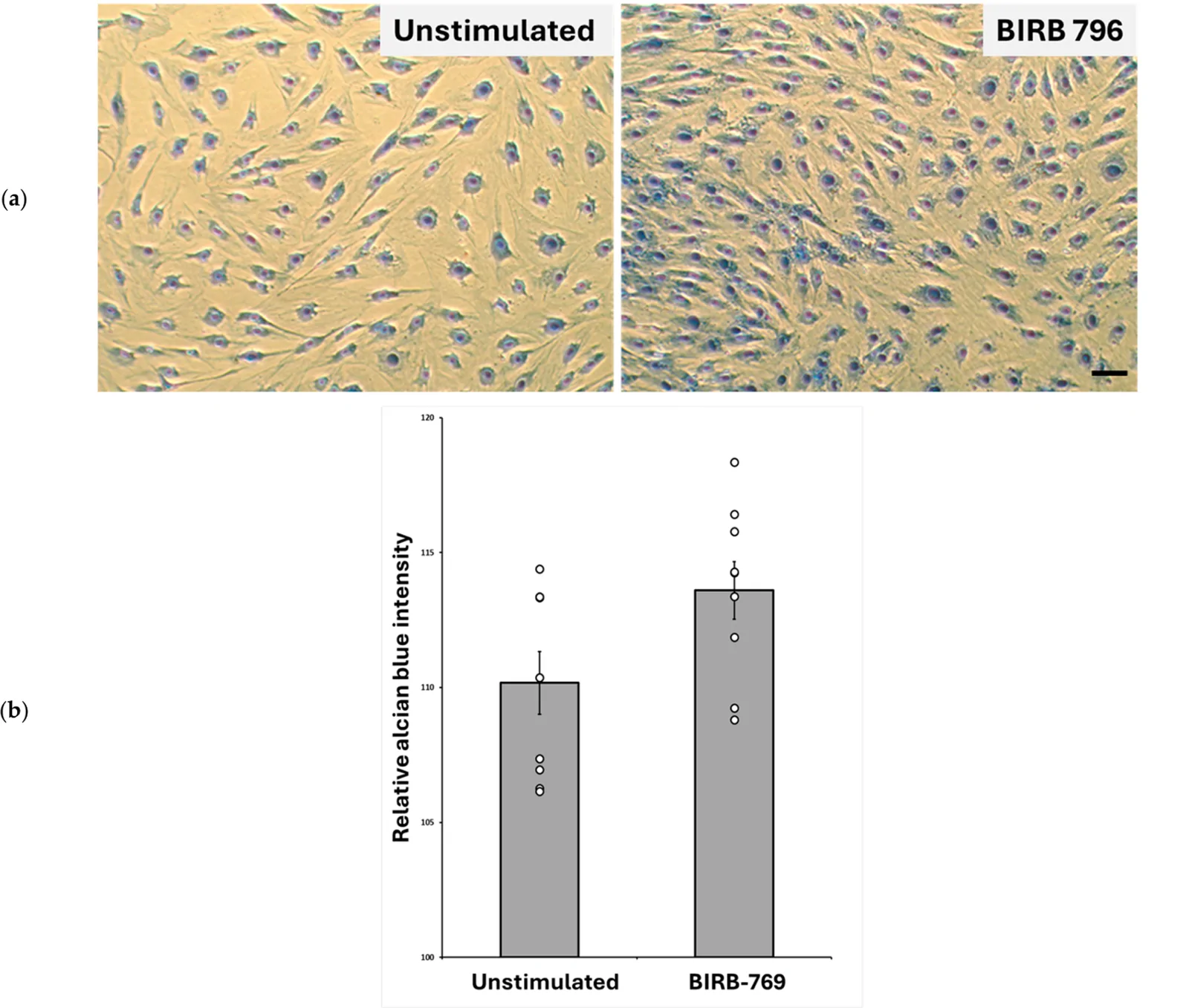
Proteoglycan synthesis increased after inhibition of p38α activity. C3H10T1/2 cells were cultured in micromasses and treated with a p38α inhibitor. (**a**) Representative image of Al-cian blue staining of C3H10T1/2 cells, Unstimulated or treated with BIRB 796 (scale bar—10 μm); (**b**) quantification of Alcian blue staining of the micromasses. The dots represent individual samples within replicates (n = 9). Alcian blue intensity increased with the treatment of p38α inhibitor in c3H10T1/2 cells compared to no inhibitor (Unstimulated) (*p* < 0.05, error bars denote standard error of mean).

Next, we studied the effect of p38α inhibition on proteoglycan synthesis. Early inhibition of p38 activates chondrogenesis in synovium-derived stem cells [32]. Inhibition of p38α activates Wnt11, which enhances chondrogenesis at later stages [32]. Reducing p38α Y182 phosphorylation reduces its activity [31]. We inhibited p38α activity using the pharmacological inhibitor BIRB796. It is a highly specific inhibitor of p38α [48]. There was a modest increase in proteoglycan synthesis in C3H101/2 cells treated with p38α inhibitor. We mimicked this by using a pharmacological inhibitor of MAPK. We observed enhanced proteoglycan synthesis (Figure 11).

## 3. Discussion

Previous research exploring the effectiveness of CK2.1 showed that it activated the SMAD-binding element in C3H10T1/2 cells upon stimulation. The extent of upregulation of proteoglycan synthesis with CK2.1 at 100 nM concentration was comparable to BMP-2 stimulation at 40 nM. CK2.1, when injected intravenously in healthy C57BL/6 mice, activates expression of chondrogenic markers such as Sox9 and Collagen type II. It also prevents the activation of chondrocyte hypertrophy markers, such as Collagen Type X and Osteorix. Moreover, in the spontaneous OA model induced by destabilized medial meniscus (DMM) surgery, CK2.1 upregulates chondrogenic markers while preventing the expression of chondrocyte hypertrophy markers. The damage to the articular cartilage was also repaired in the DMM mice [13,24]. However, the underlying mechanism of these effects of CK2.1 has not been studied before. Thus, we used a phosphoproteomics-based approach, together with total protein expression, to study the mechanism of CK2.1.

We used the C3H10T1/2 cells as a model because they are well-suited to studying chondrogenic differentiation. These cells undergo chondrogenic differentiation when cultured in micromasses and treated with BMP-2 [49–51]. The protein expression profile is relatively well characterized. Also, it is easier to obtain a high total protein amount from a uniform cell line, which is required for a phosphoproteomics study. We validated the results in the CK2.1 mouse model injected intravenously. Our previous research showed that systemic injection of CK2.1 effectively restores articular cartilage without inducing chondrocyte hypertrophy [52]. Thus, we used the same model to validate differential phosphorylation. This validation helps extend the previously known effects of CK2.1 in vivo. Also, these samples have been harvested after longer treatment with CK2.1. The sustained differential phosphorylation of the markers establishes better applicability of the phosphoproteome. Additional analysis of differential phosphorylation of the same phosphosites in C3H10T1/2 cells with Western blotting or immunofluorescence would have added more direct validation.

We predicted that overlapping phosphosites, differentially phosphorylated by BMP-2 and CK2.1, would activate chondrogenesis. This was due to their convergent effects on chondrogenic differentiation of C3H10T1/2 cells [24]. BMP-2 activates chondrocyte hypertrophy in articular chondrocytes [9,53,54]. We predicted that the phosphosites exclusively differentially phosphorylated in BMP-2-treated cells would activate chondrocyte hypertrophy. Those exclusively differentially phosphorylated in the CK2.1-treated groups were expected to activate chondrogenic differentiation. Based on the PLS-DA analysis, the lack of overlap between the lists indicated that the mechanism by which CK2.1 activates chondrogenesis is distinct from that of BMP-2. However, the identified phosphosites require mechanistic validation before their significance in chondrogenesis can be established. We predicted a mechanism by which CK2.1 activates chondrogenesis. The future work from this study should focus on validating the mechanism in greater detail.

The BMP signaling pathway plays a significant role during cartilage development and homeostasis. In OA, there is upregulation of BMP-2 in synovial fluid and articular cartilage [55]. There is a positive correlation between BMP-2 expression levels and OA disease progression [55]. Articular chondrocytes express higher levels of Bmpr1a in OA patients [56]. The BMP-2 also activates osteoblastic differentiation of chondrocytes by increasing Alkaline phosphatase activity [10]. BMP-2 is essential for maintaining chondrocyte phenotype. However, sustained BMP-2 stimulation activates Osteopontin and Runx2 expression [57]. In prehypertrophic and hypertrophic cartilage zones, similar signaling pathways are activated, leading to upregulation of osteoblastic differentiation factors, such as RunX2, and the production of bone-specific collagens, including collagen type X. In fact, hypertrophic differentiation during OA is a similar process to endochondral ossification. The canonical BMP signaling via the Smad pathway is upregulated in severe OA [14,51], specifically in regions of articular cartilage where ectopic ossification is observed [58]. Smad-dependent BMP signaling is required for endochondral ossification [59].

The noncanonical BMP signaling MAPK pathway coordinates with the canonical signaling to help regulate the differentiation of chondrocytes [60]. Of the three MAPKs, ERK, p38, and JNKs, the latter two are activated in response to inflammatory signals. The p38 MAPK signaling induced by BMP2 activates ALP activity and drives osteoblastic differentiation [31]. On the contrary, p38α stabilizes Sox9 mRNA [30]. Sox9 is a pivotal transcription factor for chondrogenic differentiation and cartilage synthesis [61]. Additionally, p38α is essential for preventing chondrocyte hypertrophy in vivo by stabilizing Sox9 expression and activity [30,32]. However, its early inhibition primes synovial stem cells towards chondrogenic fate [32]. Thus, the role of p38α in chondrogenesis is more complex than just an activator or inhibitor [32].

In this analysis, we propose a molecular mechanism by which CK2.1 induces chondrocyte genesis and cartilage synthesis. The differential phosphor-proteome between CK2.1 and BMP-2 was captured along with differentially expressed proteins. This approach helps us analyze a larger number of phosphosites to generate hypotheses. The robustness of the analysis was assessed using supervised clustering with PLS-DA. The clear distinction between BMP-2-induced and CK2.1-induced chondrogenic activation indicates that these activations depend on distinct sets of phosphosites. The list of differentially phosphorylated proteins includes Fkbp15, which is a Bmpr1a-anchored protein. It is a cytoplasmic protein belonging to the FK506-binding protein class. These proteins interact with the intracellular domain of Bmpr1a and modulate BMP pathway activation. Inhibition of their interaction with Bmpr1a by the drug FK506 activates ligand-independent BMP pathway activation [21,62]. The Fkbp15 protein inhibits BMP signaling by binding to the receptor. Its upregulated phosphorylation in CK2.1-treated samples indicates that BMP signaling is active. Similarly, Stk3 phosphorylation is downregulated. The kinase is part of the Hippo pathway. The Stk3/4 proteins are upstream of apoptotic pathway activation [25,63]. Their downregulation in CK2.1-treated samples suggests a possible mechanism by which CK2.1 inhibits chondrocyte hypertrophy. Since chondrocyte hypertrophy requires activation of apoptotic pathways, other proteins, such as Prkd1, Nol8, and Pom121, are localized to the nucleolus. Protein kinase D1/(Prkd1) is a downstream activator of the mTORC1 complex [26]. Deletion of mTORC1 rescues the OA phenotype in the DMM mouse model [27]. The mTORC1 complex regulates autophagy and apoptosis in chondrocytes [28]. Their involvement could be further investigated in relation to CK2.1’s regulation of cell fate. Nol8 binds the β-catenin protein in the nucleus. The binding of Nol8 results in the modulation of canonical Wnt/β-catenin-regulated gene expression [18]. The Wnt/β-catenin pathway is essential for cartilage repair post-injury [19]. Pom121 is a nucleolar protein. Pom121 overexpression upregulates PI3K/Akt signaling [64]. Inhibition of the PI3K/Akt signaling pathway reduces chondrocyte hypertrophy [65].

The phosphoproteomics approach helped identify activation/inhibition of specific pathways caused by changes in phosphorylation levels. The phosphoproteomics and total proteomics approaches provide snapshots of phosphorylation and protein expression. However, the direction of regulation (upstream regulators and downstream targets) is not established solely by these analyses. Additionally, we only tested the phosphorylation and total protein at a single time point after CK2.1 and BMP-2 treatment. Thus, we cannot infer later-stage events from current data. Chondrogenesis and upregulation of proteoglycans occur over a longer period [66,67]. Phenotypic stability of chondrocytes is also an essential factor in the success of chondrogenesis-inducing treatments. With our current analysis, we did not study that aspect. However, we have not characterized other post-translational modifications than phosphorylation. We did not study protein–protein interactions with this approach either. We used a uniform cell line to amplify total protein levels during early chondrogenic induction. However, in vivo, there are three different types of chondrocytes in the articular cartilage zones: superficial, middle and deep zone chondrocytes. These are metabolically and functionally distinct classes. In the present study, we did not study responses of these different types of chondrocytes to CK2.1.

The list of proteins differentially phosphorylated by CK2.1 compared with Unstimulated C3H10T1/2 cells showed no connections to canonical BMP-2 signaling in the STRING database network analysis. A few MAPK pathway proteins were on the list, and other proteins on the list were associated with the MAPK pathway. The BMSCs derived from 6-month-old and 15-month-old mice also showed an association with MAPK signaling proteins. The MSCs arising from BMSCs are an important source of pre-chondrogenic cells. Thus, BMSCs have been explored for use in autologous transplants for chondrocyte replacement therapies [68]. Moreover, their role in regulating inflammation during OA progression is significant [6]. The MAPK pathway is essential for regulating several pathways downstream of inflammatory cytokines [69]. Thus, MAPK differential activation in BMSCs becomes even more substantial in understanding the pathogenesis of OA. STRING database is a predictive tool. The association of proteins based on network analysis in STRING is not equivalent to the characterization of a molecular pathway or protein–protein interactions. Additionally, the analysis is not specific to chondrocytes.

The p38 Y182 phosphosite was among the sites with reduced phosphorylation. Phosphorylation of p38 at Y182 is required for its activation. Compared to non-p38-inhibited cells, p38-inhibited cells showed an increase in proteoglycan synthesis, assessed by Alcian blue staining. Increased proteoglycan synthesis occurs during chondrogenic differentiation of C3H10T1/2 cells. Thus, we predicted a mechanism in which CK2.1 inhibits p38 activity, thereby inducing cartilage synthesis.

## 4. Materials and Methods

### 4.1. Sample Preparation

Mouse mesenchymal C3H10T1/2 cells were purchased from ATCC. Cells were cultured using high-glucose DMEM supplemented with Sodium pyruvate and Glutamine. Anti-antimycotic and Penicillin-Streptomycin were added, and the media were supplemented with 10% FBS. Cells were grown in a T75 flask for 75% confluence. Cells were treated with FBS-free DMEM for 24 h to serum starve. Cells were treated with CK2.1 (100 nM), BMP2 (40 nM), or left untreated as an Unstimulated control. After 6 h of treatment, cells were harvested by washing with PBS and scraping them into 1 mL of PBS. The cell suspension was centrifuged at 1000 rpm for 10 min at 4 ^◦^C. Supernatant was removed after centrifugation. Total cell pellets were stored and shipped to UAMS over dry ice.

### 4.2. Mouse BMSC Harvesting

C57BL/B6 mice aged 6 and 15 months (n = 9 each) were obtained from the Nathan Shock Center (San Antonio, TX, USA). Upon arrival at the Life Science Research Facility (University of Delaware, Newark, DE, USA), the mice were housed in groups of 4 or 5 per cage and allowed to acclimatize for 1 week. The mice were weighed prior to any experimentation. Water and food were available to the mice throughout this study. This research study was reviewed and approved by the Institutional Animal Care and Use Committee (University of Delaware, Newark, DE, USA): AUP #1194.

Bone marrow stromal cells were isolated from the left femurs of the mice. First, the distal and proximal femoral heads were removed from the femurs to expose the bone marrow. Next, the bones were flushed with alpha minimum essential media (αMEM; Caisson Labs, Smithfield, UT, USA), and the solution was filtered with a 70 μM cell strainer (Stellar Scientific, Baltimore, MD, USA) into 50 mL conical tubes (Cole-Palmer, Vernon Hills, IL, USA). The cells were then centrifuged at 1500 rotations per minute (rpm) for 5 min at 4 ^◦^C and resuspended in 5 mL of αMEM.

### 4.3. BMSC Culture and Treatment with CK2.1

BMSCs were seeded in a T175 flask at a density of 3 × 10^7^ cells per flask. Cells were incubated for 4 days at 37 ^◦^C and 5% CO_2_. On day 5, cells were serum-starved for 24 h. Afterward, cells were treated with 100 nM CK2.1 for 6 h. The cell pellet was isolated by centrifugation for 10 min at 4 ^◦^C at 1500 rpm.

### 4.4. Mouse Injections

Mouse injections were performed as a tail vein injection protocol described in ‘CK2.1, a novel peptide, induces articular cartilage formation in vivo.’ [13,24] and an intra-articular injection protocol described in ‘CK2.1, a Bone Morphogenetic Protein Receptor Type Ia mimetic peptide, repairs cartilage in mice with destabilized medial meniscus’.

### 4.5. Protein Extraction and Mass Spectrometry

Total protein from each sample was reduced, alkylated, and purified by chloroform/methanol extraction before digestion with sequencing-grade modified trypsin/LysC (Promega, Madison, WI, USA). Tryptic peptides were labeled using a tandem mass tag 10-plex isobaric label reagent set (Thermo, Waltham, MA, USA) and enriched using High-Select TiO_2_ and Fe-NTA phosphopeptide enrichment kits (Thermo) following the manufacturer’s instructions. Both enriched and un-enriched labeled peptides were separated into 46 fractions on a 100 × 1.0 mm Acquity BEH C18 column (Waters, Milford, MA, USA) using an UltiMate 3000 UHPLC system (Thermo) with a 50 min gradient from 99:1 to 60:40 buffer A: B ratio under basic pH conditions and then consolidated into 18 super-fractions. Each super-fraction was then further separated by reverse phase XSelect CSH C18 2.5 um resin (Waters) on an in-line 150 × 0.075 mm column using an UltiMate 3000 RSLCnano system (Thermo). Peptides were eluted using a 75 min gradient from a 97:3 to a 60:40 buffer A: B ratio. Eluted peptides were ionized by electrospray (2.4 kV) followed by mass spectrometric analysis on an Orbitrap Eclipse Tribrid mass spectrometer (Thermo) using multinotch MS3 parameters. MS data were acquired using the FTMS analyzer in top-speed profile mode at a resolution of 120,000 over a range of 375 to 1500 *m*/*z*. Following CID activation with a normalized collision energy of 31.0, MS/MS data were acquired using the ion trap analyzer in centroid mode and normal mass range. Using synchronous precursor selection, up to 10 MS/MS precursors were selected for HCD activation with a normalized collision energy of 55.0, followed by acquisition of MS3 reporter ion data using the FTMS analyzer in profile mode at a resolution of 50,000 over a range of 100–500 *m*/*z*. (Buffer A = 0.1% formic acid, 0.5% acetonitrile, Buffer B = 0.1% formic acid, 99.9% acetonitrile; both buffers adjusted to pH 10 with ammonium hydroxide for offline separation).

### 4.6. Data Processing

Proteins were identified and reporter ions quantified by searching the UniprotKB database restricted to Mus musculus (February 2023) using MaxQuant (Max Planck Institute, version 2.2.0.0) with a parent ion tolerance of 3 ppm, a fragment ion tolerance of 0.5 Da, a reporter ion tolerance of 0.001 Da, trypsin/P enzyme with 2 missed cleavages, variable modifications including oxidation on M, Acetyl on Protein N-term, and phosphorylation on STY, and fixed modification of Carbamidomethyl on C. Protein identifications were accepted if they could be established with less than 1.0% false discovery. Proteins identified only by modified peptides were removed. Protein probabilities were assigned by the Protein Prophet algorithm [70]. TMT MS3 reporter ion intensities are analyzed for changes in total protein in the unenriched lysate sample. Phospho (STY) modifications were identified using the samples enriched for phosphorylated peptides. The enriched and un-enriched samples are multiplexed using two TMT10-plex batches, one for the enriched and one for the un-enriched samples.

Following data acquisition and database search, the MS3 reporter ion intensities were normalized using ProteiNorm (https://github.com/ByrumLab/proteiNorm, accessed on 3 June 2026) [71]. The data were normalized using Variance Stabilization Normalization (VSN) [72] and analyzed using proteoDA (https://github.com/ByrumLab/proteoDA, accessed on 3 June 2026), with Linear Models for Microarray Data (limma) (https://bioconductor.org/packages/release/bioc/html/limma.html, accessed on 3 June 2026) and empirical Bayes (eBayes) (http://www.dkfz.de/abt0840/whuber, accessed on 3 June 2026) smoothing of the standard errors [73,74]. A similar approach is used for differential analysis of phosphopeptides, with a few additional steps. The phosphosites were filtered to retain only peptides with a localization probability > 75%, to filter peptides with zero values, and to log2-transform. Limma was also used for differential analysis. Proteins and phosphopeptides with *p*-value < 0.05 were considered significant.

### 4.7. Bioinformatics Analysis and Data Visualization

#### 4.7.1. Supervised Clustering

We filtered all phosphosites with *p*-values < 0.05 (see Supplementary Materials Figure S1). The list of phosphosites, along with VSN-normalized intensity values, was used to perform PLS-DA analysis in MetaboAnalyst 6.0 (https://www.metaboanalyst.ca). Data normalization was already performed using the log2 VSN method. Missing values were calculated using the mean of each sample. Graphs denoting 2D plots and VIP scores were prepared using default values. For VIP loadings, the top 20 phosphosites were selected per group.

Association of phosphosites with the BMP pathway. A list of BMP pathway proteins was compiled from the literature. The keywords used were ‘BMP2’, SMAD activation’, and ‘Noncanonical BMP signaling’. The lists are provided in the Supplementary Materials (Table S1).

All the proteins with intracellular function were selected. From the list of phosphosites differentially phosphorylated by CK2.1 (*p* < 0.05), a list of protein symbols was created using the Uniprot database. The combined lists were loaded into the STRING database (https://string-db.org/). For the network analysis, connections based on ‘text mining’ were excluded. Non-connected nodes were removed. For mouse BMSCs, nodes with only high confidence were selected in the visualization due to the greater number of differentially phosphorylated proteins.

#### 4.7.2. Immunofluorescence

Deparaffinization was carried out with two washes of Xylene, followed by decreasing concentrations of Ethanol (100, 95, 90, and 70% *v*/*v*). Antigen retrieval was performed by incubating the explants in 10% *w*/*v* Bovine testicular hyaluronidase in 3% BSA solution at 37 degrees Celsius. Explants were incubated overnight with a 1:100 dilution of primary antibodies. Phospho STK3 was labeled using MST4/MST3/STK25 (T178/T190/T174) Antibody, (Catalog number bs-10441R-BF488, Bioss, AbBy Fluor^®^ 488 Conjugated, Woburn, MA, USA) and phospho PRKD1 was labeled using Anti-Phospho PKD1/2/3/PKC mu (Ser738 + Ser742) PRKD1 Antibody (Boster bio Catalog number -P30439, Pleasanton, CA, USA). The next day, explants were washed with 1X PBS. The PRKD1-stained explants were then incubated in a 1:500 dilution of the secondary antibody Alexa 488 for 1 h. Nuclei were stained with Hoechst (5 mg/mL in 10 mL) for 15 min and washed twice with 1X PBS for 15 min each.

#### 4.7.3. Micromass Culture

Mouse mesenchymal C3H10T1/2 cells were purchased from ATCC (cat no. CCL-226). Cells were cultured using high glucose DMEM (Millipore Sigma, cat no. D7777–10L, Burlington, MA, USA) supplemented with Sodium pyruvate and Glutamine. Anti-antimycotic (Hyclone cat no. SV30079.01, Logan, UT, USA) and Penicillin-Streptomycin (Gibco cat no.15140122, Thermo Fisher Scientific) were added, and the media were supplemented with 10 percent FBS (Gemini, cat no. 100–106-500, West Sacramento, CA, USA). Cells were grown in a T75 flask for 75% confluence. Cells were concentrated to obtain 10^7^ cells in 10 μL of DMEM. A 10 μL volume was placed in the center of each well in a 12-well plate. The cells were incubated at 37 ^◦^C and 5% CO_2_ for 2 h to adhere to the well plate surface. Later, 1 mL of fresh complete DMEM (DMEM supplemented with 10% FBS) was added to each well.

#### 4.7.4. MAPK Inhibitor Treatment

The micromasses were treated with Doramapimod (BIRB 796) 76 nM (double the IC50). The treatments lasted 1 week.

#### 4.7.5. Alcian Blue Staining

Micromasses were washed with phosphate-buffered saline (PBS) thrice. Micromasses were fixed with formalin-PCP (pH 7.4) for 30 min. Then, they were treated with glacial acetic acid for 30 min. Micromasses were stained using Alcian blue dye (pH 2.5) for 24 h. Afterward, micro masses were washed with glacial acetic acid for 24 h. Finally, plates were dried at room temperature for 48 h.

#### 4.7.6. Quantification of Alcian Blue Staining

The intensity of Alcian blue staining was quantified using ImageJ (1.54p) [75]. The mean red intensity was measured from a 100 × 100-pixel circular region centered on each stained micromass. These values were all subtracted from 255.

To remove the background, the background for each well was determined by averaging 4, 60 × 60-pixel square red-intensity selections—one selection in each corner, avoiding the well edges and the micromass itself. These values were averaged for each well and subtracted from 255. For each well, the 255-mean background was subtracted from the 255-mean red intensities of the cells. This provides the staining intensity value for each well.

## 5. Conclusions

Osteoarthritis is a disease of complex etiology. Specifically, spontaneous or age-induced OA has aging and inflammatory components in most patients [68]. The older description of OA was that it was an inflammatory form of arthritis. Recent changes in perspective on OA characterization now describe it as a metabolic disease. This has led to an expansion of our understanding of the molecular signaling pathways underlying the symptoms—synovial inflammation, cartilage degeneration, and osteophyte formation [5]. The developmental pathways in joint development could be leveraged to develop an effective therapeutic for OA.

Phosphoproteomics analysis provides a snapshot of cellular protein phosphorylation. We utilized this analysis to characterize the distinct activation or deactivation of cellular pathways by BMP-2 and CK2.1. We identified a set of differentially phosphorylated phosphosites specific to both. We also analyzed the interactions between BMP signaling components—canonical and non-canonical with the identified phosphosites. In CK2.1-treated samples, MAPK proteins were differentially phosphorylated and were connected to a few other proteins on the list. The functional relevance of these interactions needs to be investigated to better understand how CK2.1 regulates MAPK signaling.

CK2.1 treatment reduced phosphorylation of p38 at Y182 in the phosphoproteomics analysis. We inhibited p38α activity to mimic the effect of CK2.1. This increased proteoglycan synthesis. Thus, CK2.1 may depend on the downregulation of p38α phosphorylation during chondrogenic activation. However, the downstream proteins and transcription factors in the p38-downregulated pathway are not identified in the current study. A mechanistic study of transcription factors that are activated or inhibited by p38 downregulation should be conducted.

## Supplementary Material

Supplemental Data Figure S1: PLS-DA loading scores and top phosphosites in BMP-2, CK2.1 and Unstimulated cells
Figure S2: Comparison of differentially phosphorylated phosphosites by CK2.1 and BMP-2 with BMP signaling
Figure S3: Differentially phosphorylated proteins by CK2.1 in BMSCs isolated from 6-month and 15-month-old mice and Gene ontology mapping.
Table S1. P38 MAPK pathway proteins.
Table S2: List of proteins included in canonical and p38 pathway signaling.

**Supplementary Materials:** The following supporting information can be downloaded at: https://www.mdpi.com/article/10.3390/kinasesphosphatases4020016/s1

## Data Availability

Available on request to the corresponding author.
