# Supplementary material for: CK2.1 Activates Chondrogenesis by Regulation of the p38 Mitogen-Activated Protein Kinase Pathway
Source: Kinases Phosphatases. Author manuscript; Available in PMC 2026 Sep 22. (PMC13593338; doi:10.3390/kinasesphosphatases4020016)
Supplement: Supplementary file 1 [file NIHMS2192436-supplement-Supplemental_Data.pdf]

kinases and phosphatases

MDPI

Supplementary data

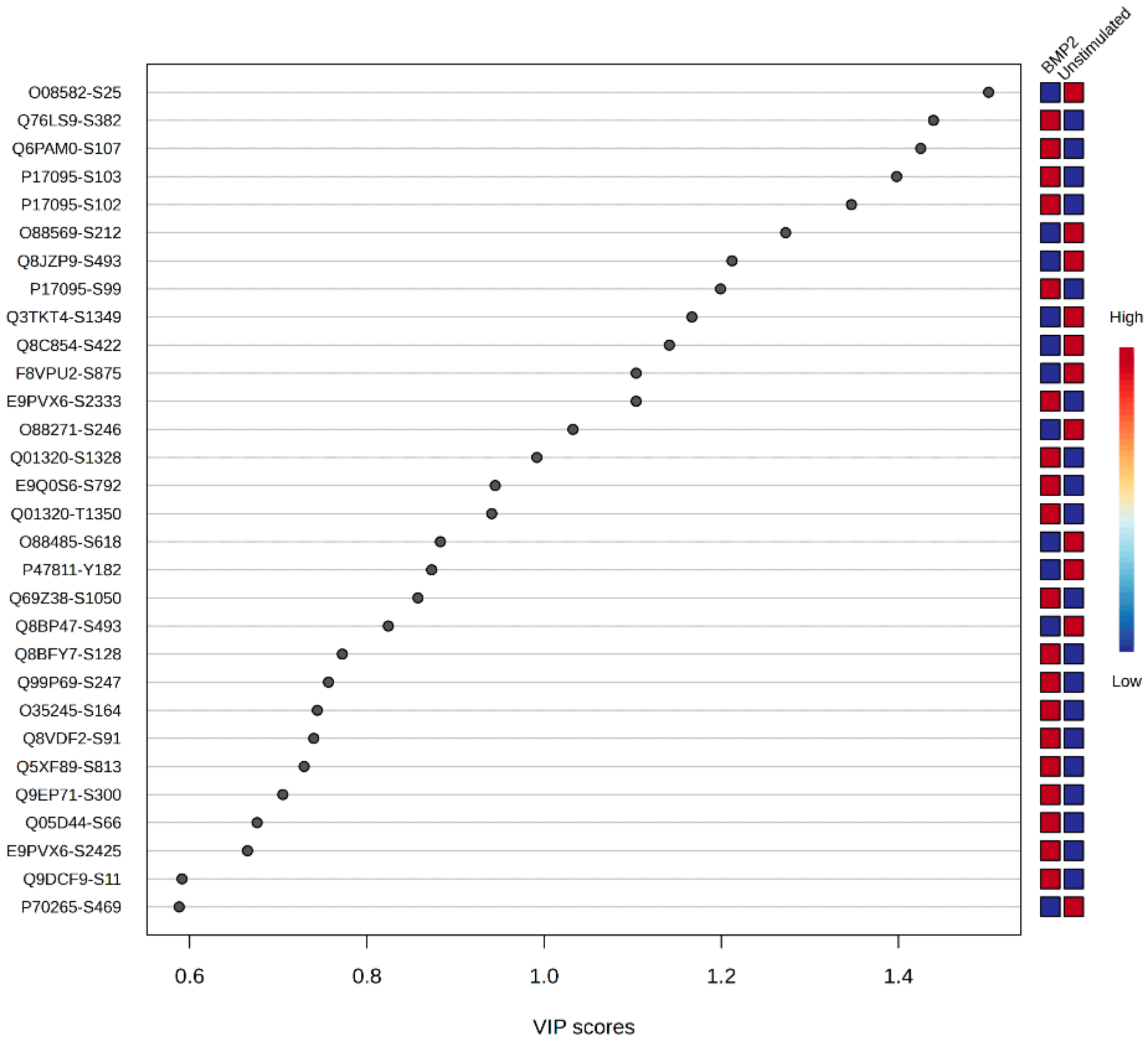

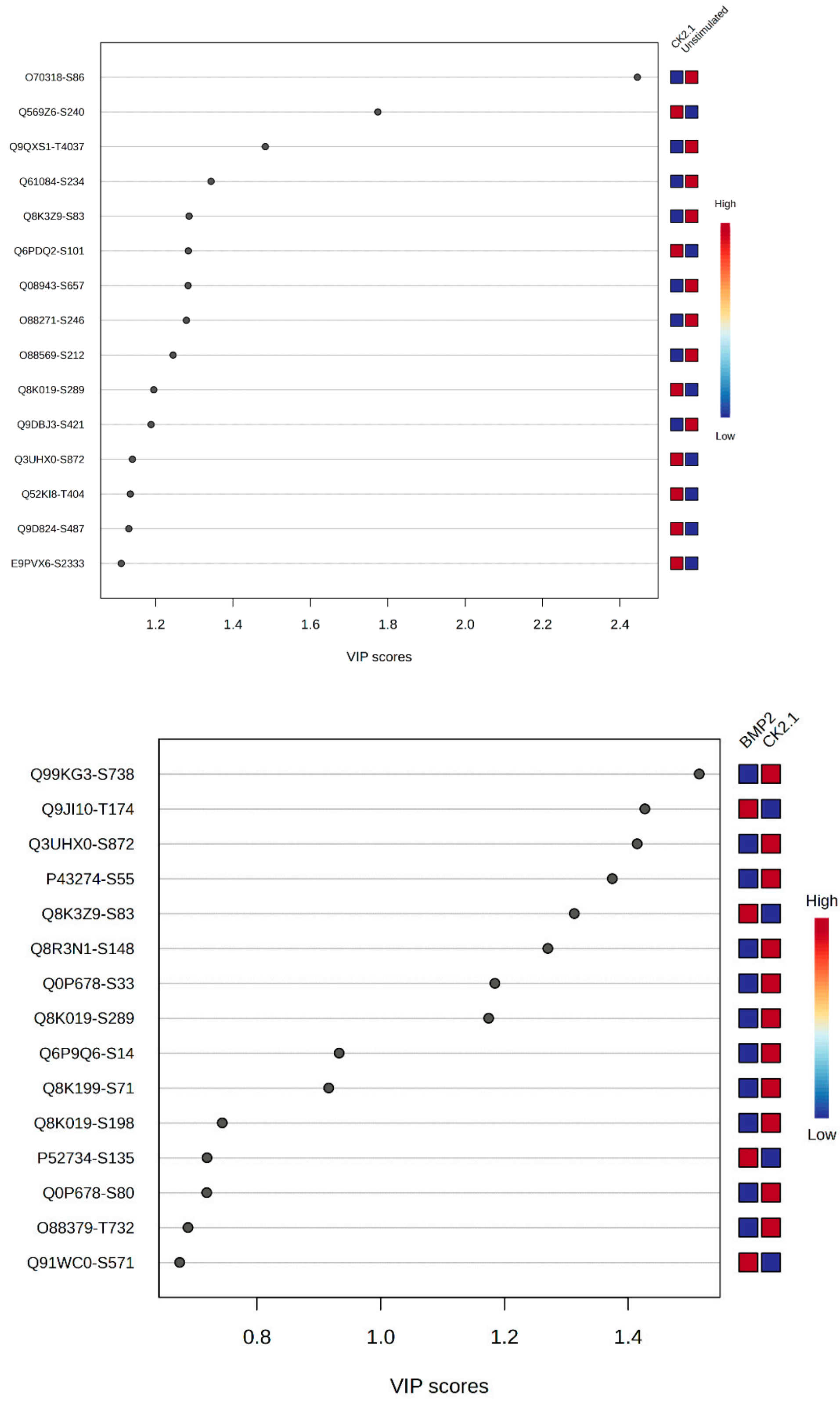

Figure S1. PLS-DA loading scores and top phosphosites in BMP-2, CK2.1 and Unstimulated cells.

BMP2 vs CK2.1

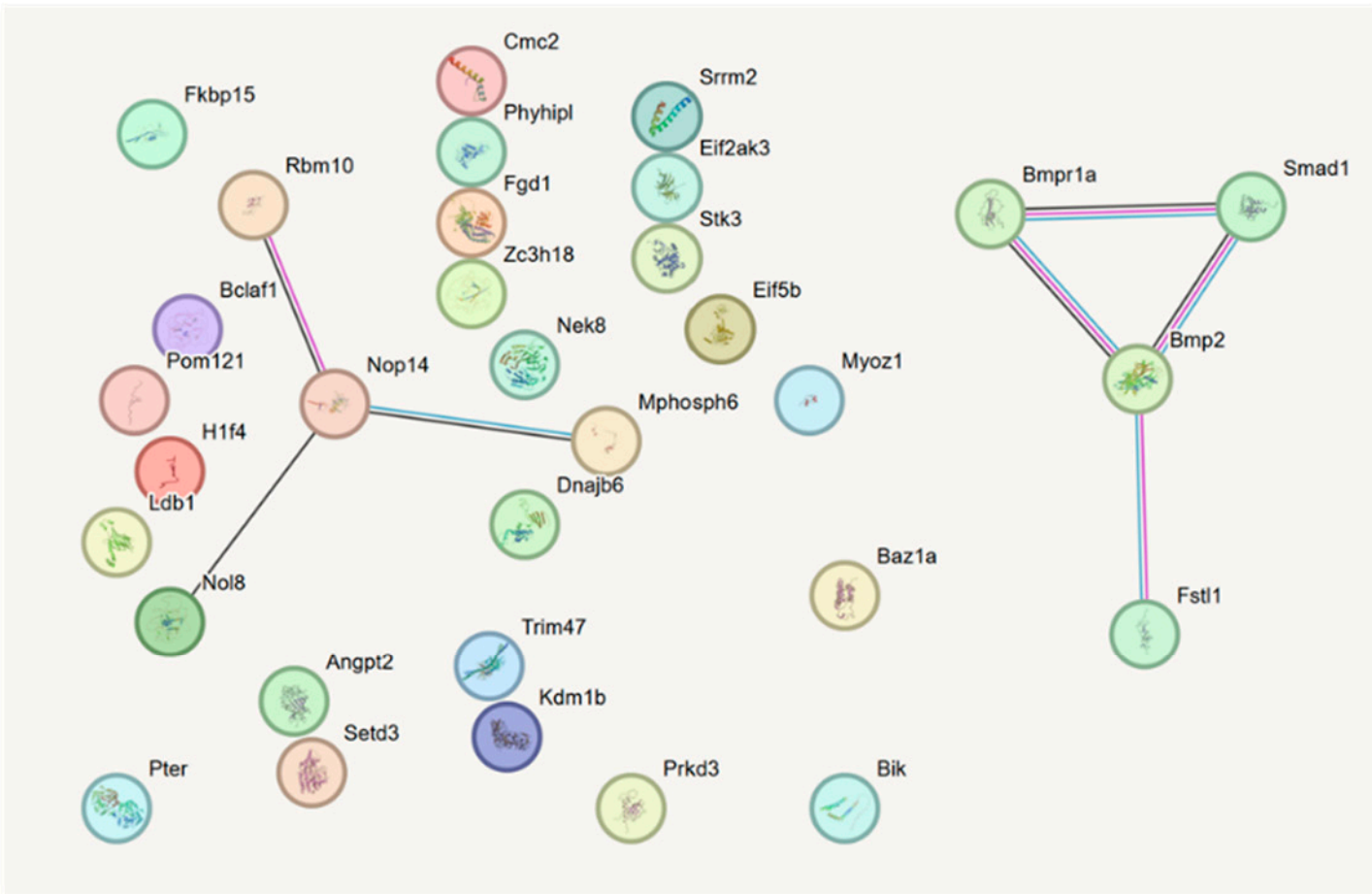

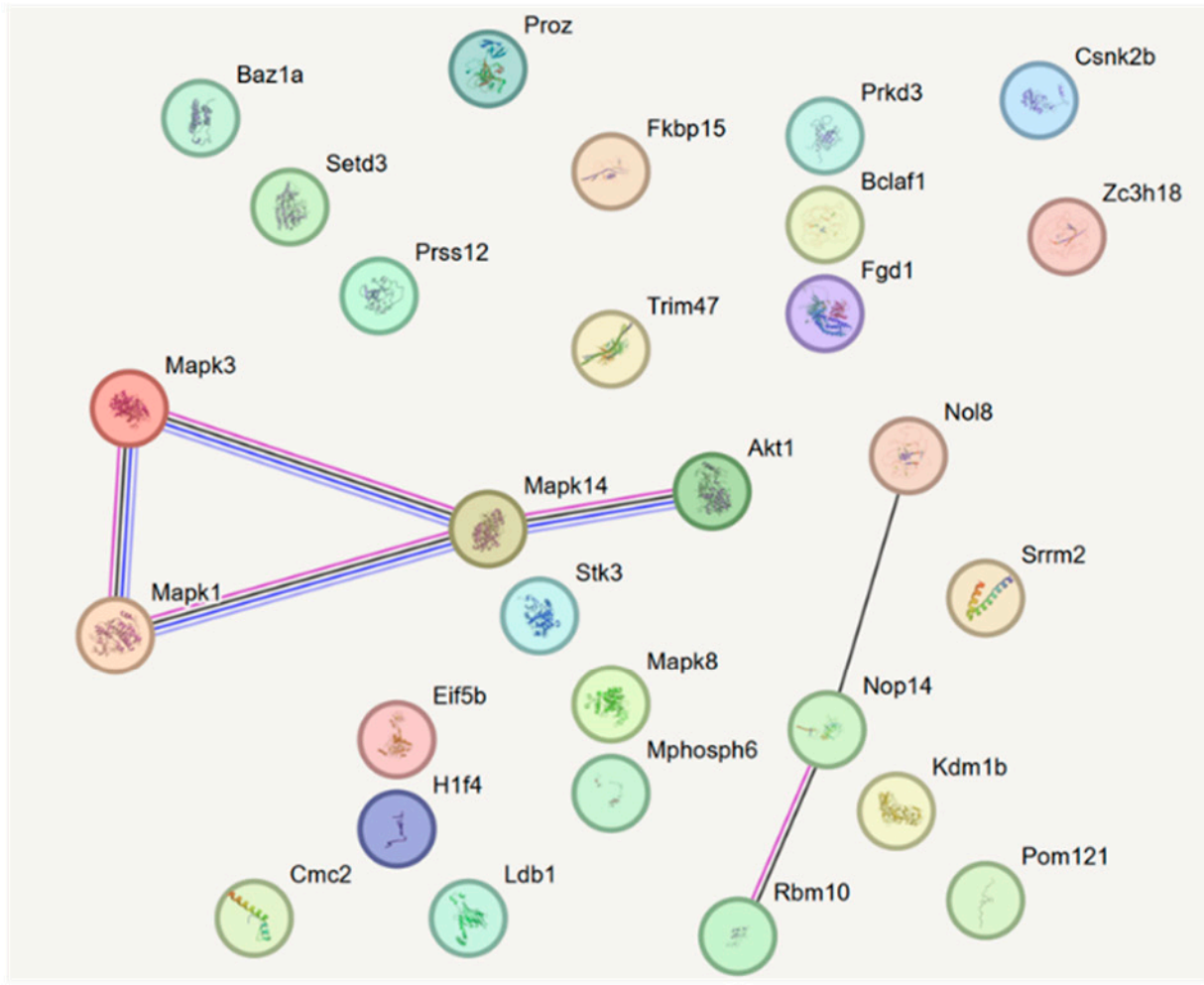

Canonical BMP signaling

Non-Canonical BMP signaling

BMP2 vsUS

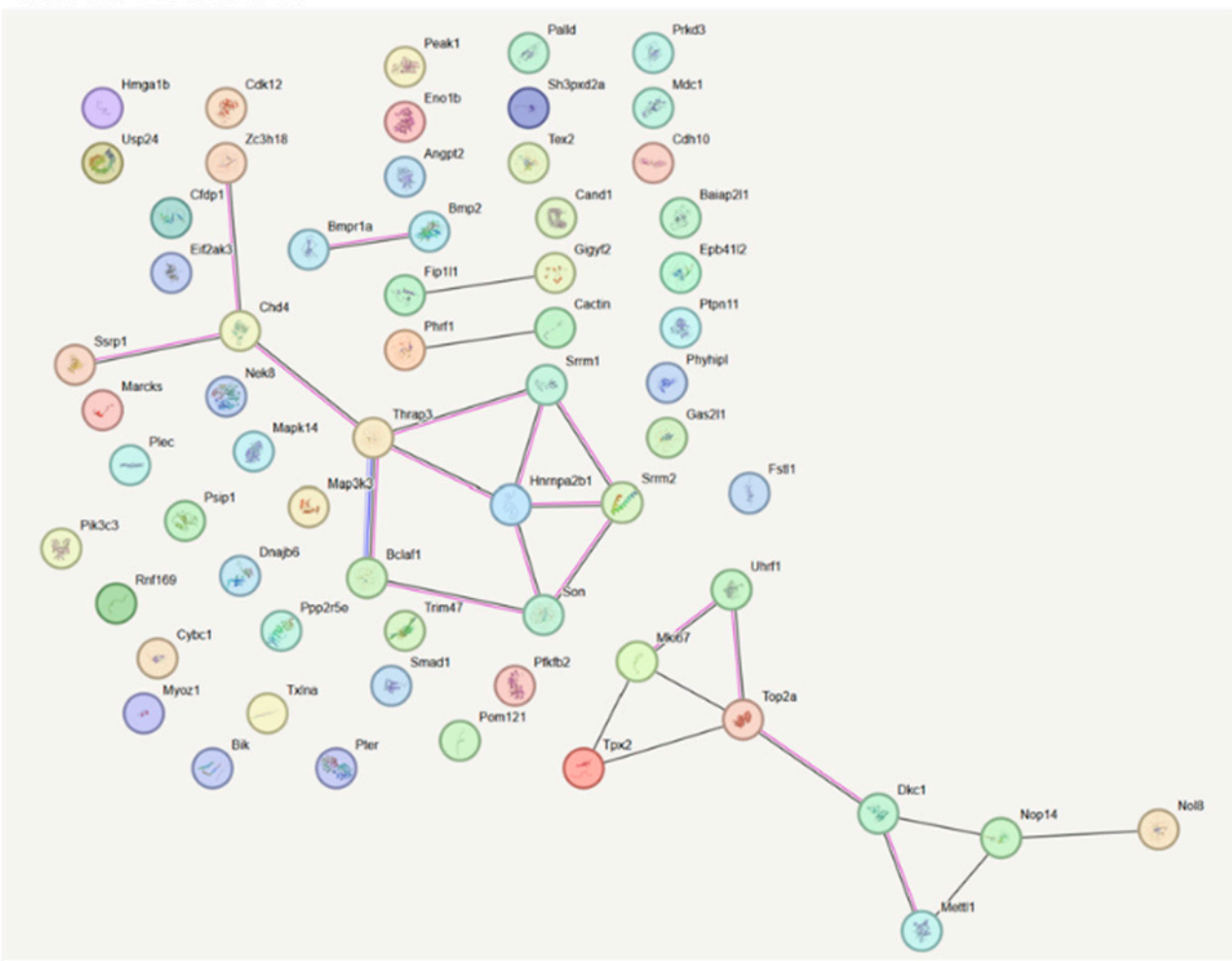

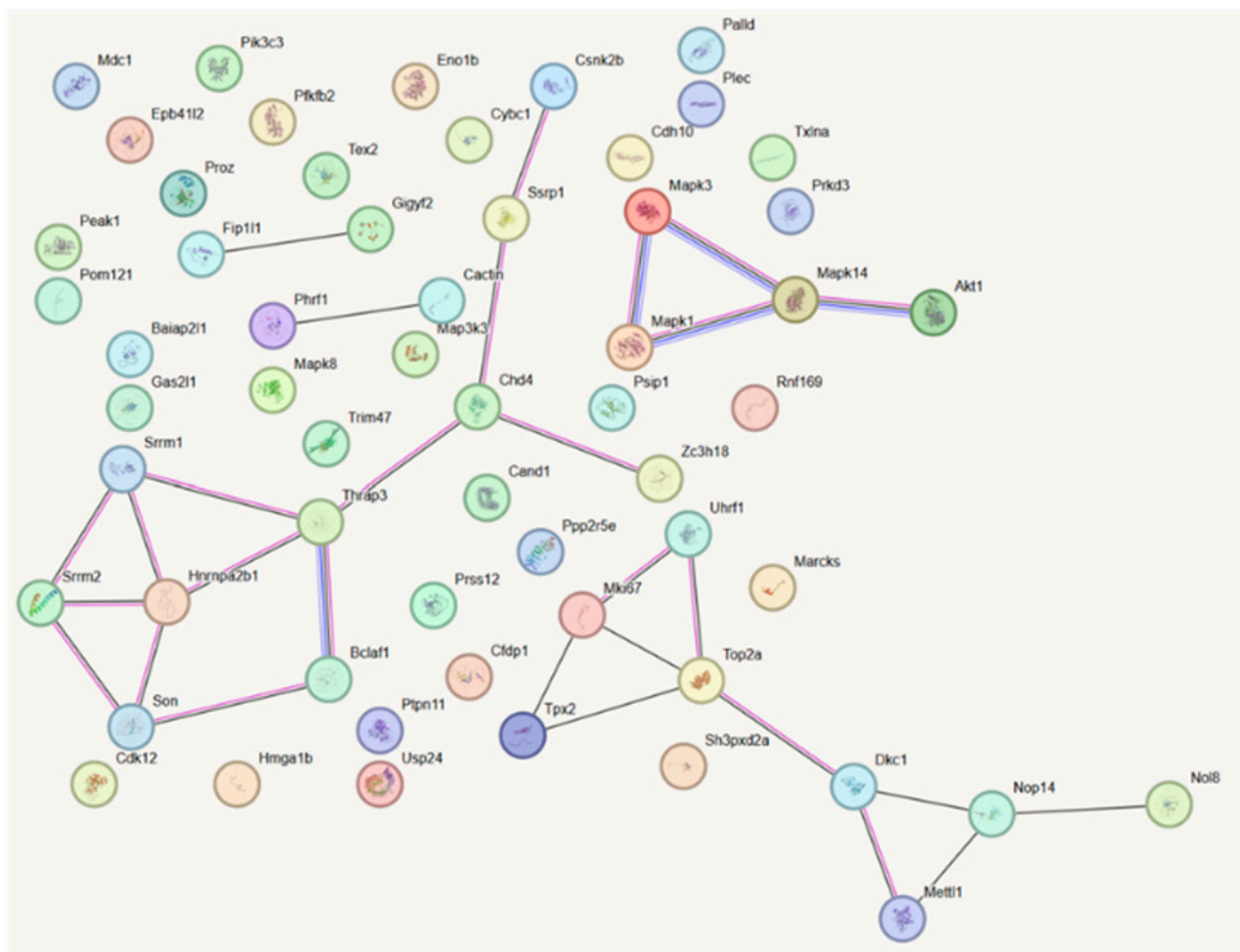

Canonical BMP signaling

Non-Canonical BMP signaling

Figure S2. Comparison of differentially phosphorylated phosphosites by CK2.1 and BMP-2 with BMP signaling.

Table S1. P38 MAPK pathway proteins.

Uniprot IDs
Q63844
P63085
P47811
Q91Y86
P49186
Q8BIM8
P31750
O55253
Q9Z2G7
Q04206
O08762
P10427
P61586
Q9CQW3
P68400
P67871
Q571K4
Q9R117
P52332
Q62120
Q00560
P08505
P22272
P35235
P63085
Q63932

Table S2. List of proteins included in canonical and p38 pathway signaling.

Uniprot IDs
P70340
P97454
Q9JIW5
P97471
O35253
O35182
P36898
P37172
P27038
P36895
O35607
P27040
P27037
Q9CUN6
A2A5Z6
Q9D0L6

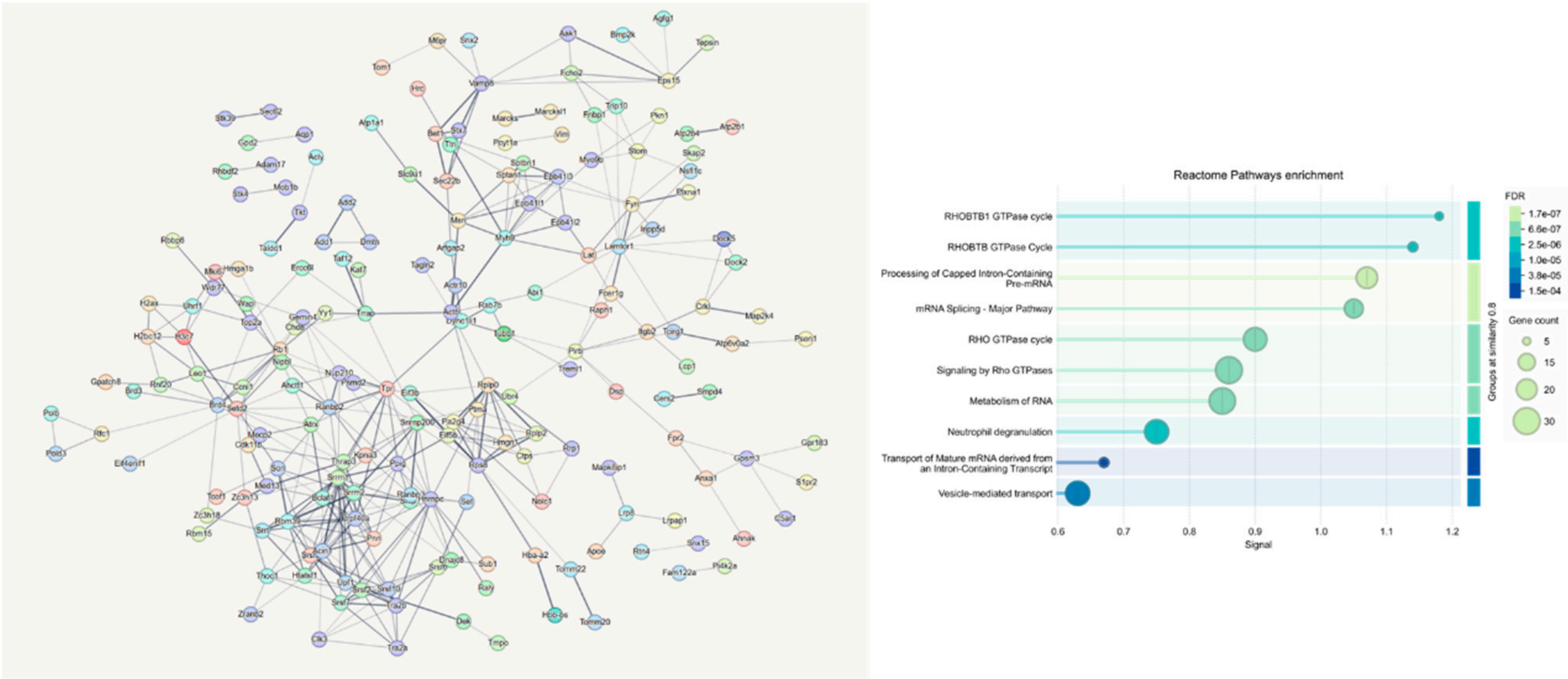

CK2.1 Phosphoproteome in 6-month-old mouse BMSCs

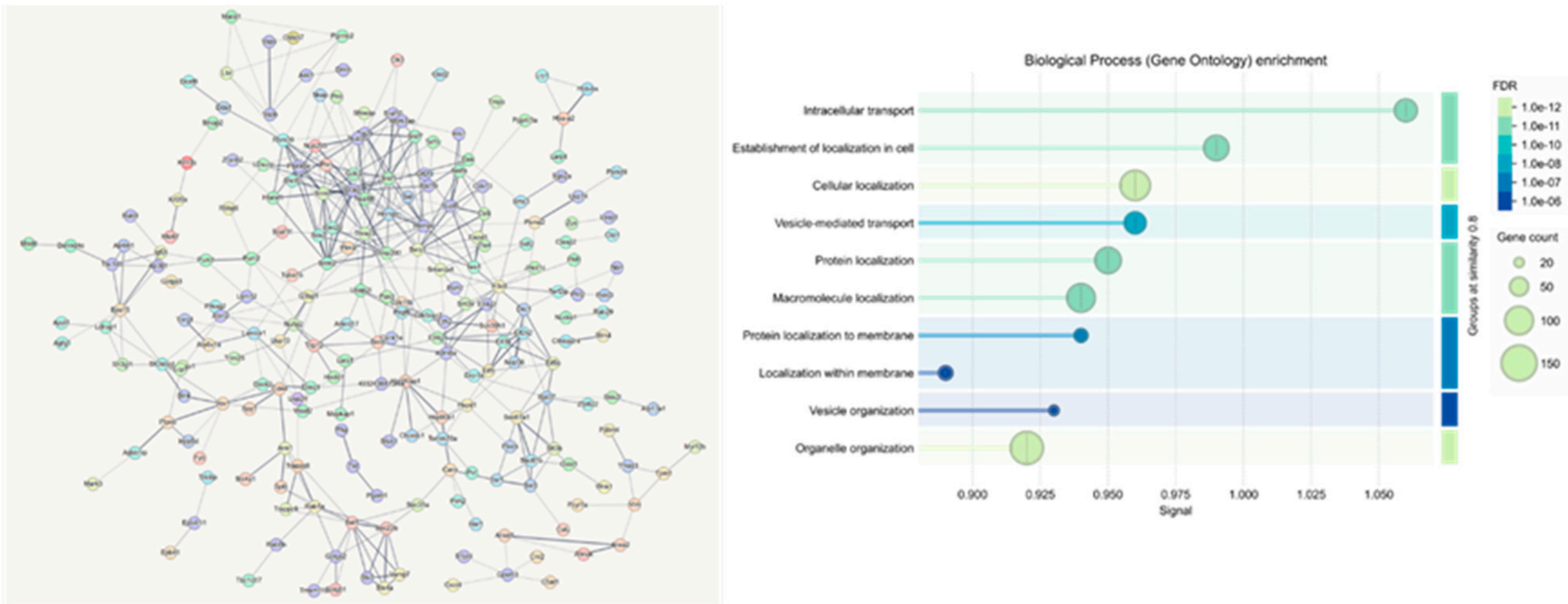

CK2.1 Phosphoproteome in 15-month-old mouse BMSCs

Figure S3. Differentially phosphorylated proteins by CK2.1 in BMSCs isolated from 6 month and 15 month old mice and Gene ontology mapping.
